# Lipid–Substrate Interactions Lead to Bilayer Asymmetry

**DOI:** 10.1021/jacs.6c04860

**Published:** 2026-05-12

**Authors:** Ruofei Wang, Ella Gregory, Brandon A. Oswald, Tinglu Yang, Paul S. Cremer

**Affiliations:** † Department of Chemistry, Penn State University, University Park, Pennsylvania 16802, United States; ‡ Department of Biochemistry and Molecular Biology, Penn State University, University Park, Pennsylvania 16802, United States

## Abstract

Lipid asymmetry was probed in supported lipid bilayers (SLBs) formed by vesicle fusion on planar glass substrates and protein-coated substrates. Leaflet distribution was determined via fluorescence microscopy following bilayer unzipping. Each SLB was primarily composed of phosphatidylcholine (PC) with 0.5 mol % of a test lipid containing a tail-labeled probe. At equilibrium, the results revealed that 75% of labeled phosphatidylserine (PS) and phosphatidylethanolamine (PE) lipids partitioned into the lower leaflet adjacent to the glass surface. This preferential partitioning further increased when experiments were conducted in D_2_O rather than an H_2_O buffer, a finding consistent with favorable hydrogen bond (H-bond) formation between surface silanols on the glass support and the headgroups of PS and PE lipids. In fact, H-bonding was the dominant factor in determining the interleaflet distribution. Varying the salt concentration could modulate the PS fraction in only the lower leaflet by a small amount, ±3%. A tail-labeled PC probe, which was unable to donate an H-bond to the surface, showed no preference for the lower leaflet once its equilibrium distribution was achieved. Finally, the 2D diffusion of the PS and PE probes, but not PC, was strongly dependent on the number of available H-bonding sites on the substrate surface. These results are reminiscent of the lipid asymmetry observed in the plasma membranes of living cells, where PS and PE are similarly partitioned into the inner leaflet, suggesting that the H-bonding environment in the cytoplasmic versus extracellular region may play a significant role in governing lipid asymmetry in vivo.

## Graphical abstract

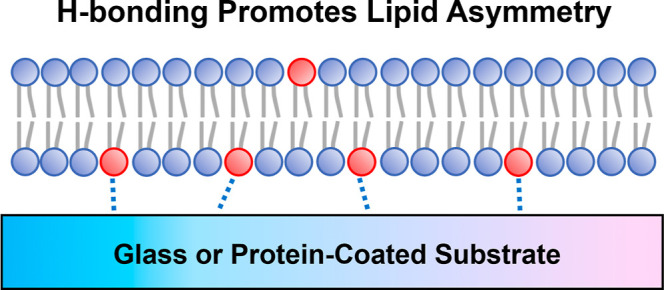

## Introduction

Plasma membranes in living cells are compositionally asymmetric, meaning the outer (exoplasmic) and inner (cytoplasmic) membrane leaflets contain different lipid populations.^1,2^ This uneven lipid distribution has important structural and functional consequences for processes such as cell signaling, apoptosis, and vesicle trafficking.^3^ Previous research has demonstrated that lipid asymmetry can be maintained, at least in part, by lipid-transport proteins, including ATP-dependent flippases and floppases, as well as ATP-independent scramblases.^4,5^ Maintaining asymmetric leaflet distributions purely though active transport would be energetically expensive, costing approximately ∼8% of a cell’s ATP budget.^6^ As such, other mechanisms are likely to be involved, which should lower the cost. One possibility concerns the differing chemical environments of the two leaflets. Specifically, the cytoplasmic side rests on the cytoskeletal network, whereas the exoplasmic leaflet faces the glycocalyx (Figure 1a).^7^ Given this intrinsic asymmetry in interfacial chemical environments, it is important to understand whether lipid–substrate surface interactions could assist in modulating the lipid partitioning between the two leaflets.

**Figure 1:**
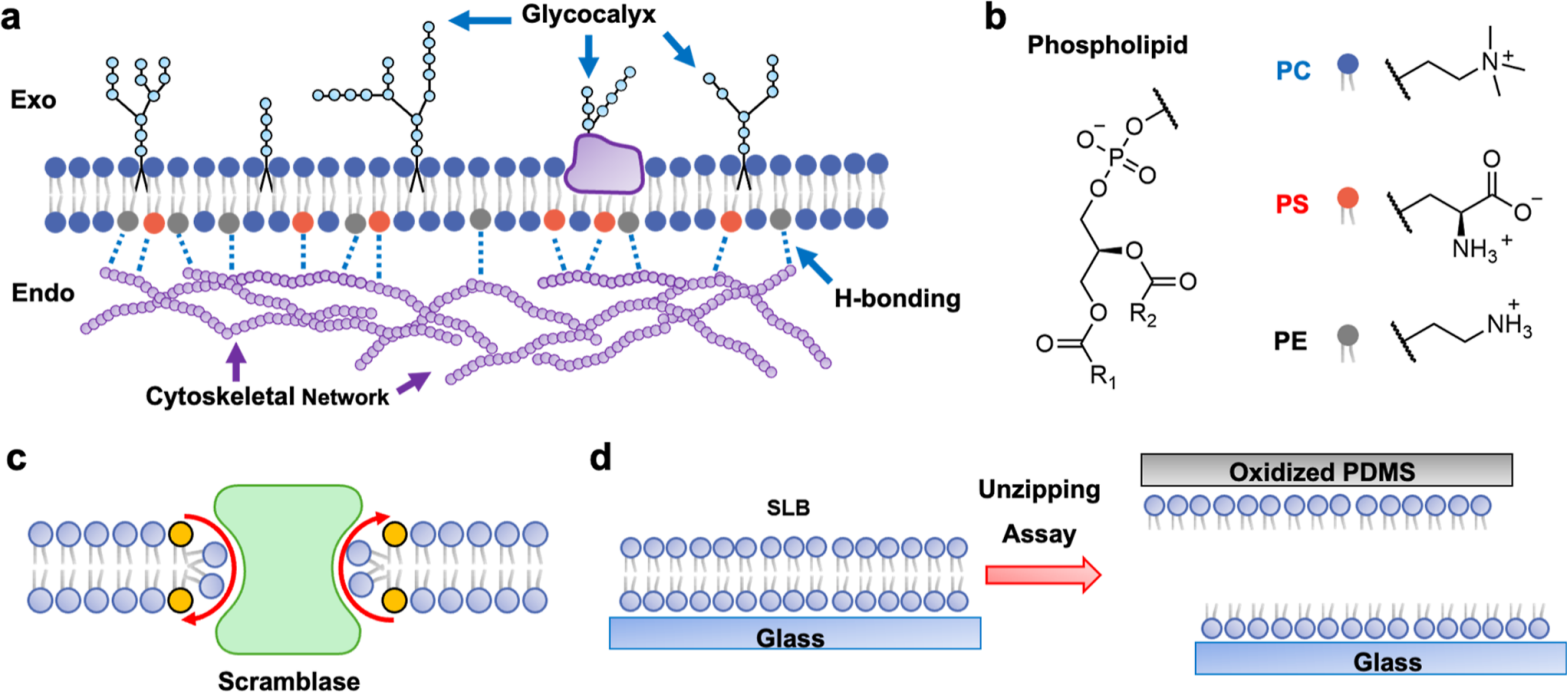
(a) Schematic illustration of lipid asymmetry in the plasma membrane of a living cell depicting H-bonding between the cytoskeleton and PS/PE lipids. (b) Chemical structures for PC, PS, and PE lipids. (c) Depiction of lipid translocation via a scramblase. (d) Schematic illustration of the lipid bilayer unzipping assay.

A striking fact of lipid asymmetry in vivo is that PS and PE are lipids primarily located on the inner leaflet of plasma membranes, while PC shows no such preference.^8,9^ Notably, both PS and PE have an amine group that can serve as an H-bond donor, while the carboxylate group on PS can serve as an H-bond acceptor (Figure 1b). By contrast, the choline moiety on PC is not capable of H-bonding. We therefore propose that weak, transient hydrogen bonding between PS/PE headgroups and the polar functional groups on proteins adjacent to the cytoplasmic interface may help bias these lipids toward the inner leaflet.

To test this hypothesis, we first examined the asymmetry of the lipid bilayers formed on borosilicate glass coverslips. In this model system, the surface silanol groups can exist in both protonated and deprotonated states,^10^ while providing a favorable hydrogen-bonding environment. Once supported lipid bilayers (SLBs) were formed via vesicle fusion,^11^ lipids were able to redistribute between the two leaflets by translocating around bilayer edge defects in a manner reminiscent of lipid scrambling in vivo that involves scramblases (Figure 1c).^12–14^

Leaflet asymmetry can be quantified using a variety of approaches.^15–22^ Here, we employed a lipid bilayer “unzipping” assay (Figure 1d).^14^ In this approach, unzipping mechanically separates the upper (bulk-solution-facing) and lower (substrate-adjacent) lipid leaflets, enabling leaflet-specific quantification by fluorescence microscopy (see Supporting Information for assay details). The results revealed that the interleaflet lipid distribution in SLBs shortly after vesicle fusion was related to both lipid partitioning in the vesicles from which supported bilayers were formed as well as lipid–substrate interactions. Significantly, the initial distributions evolved over tens of hours until equilibrium was reached. The presence of hydrogen bond donors on the lipid headgroups was the dominant factor in determining the final lipid distribution, although buffer conditions also played a modest role. Specifically, PE and PS lipids partitioned favorably into the lower bilayer leaflet, adjacent to the substrate.

Supported bilayers were also formed on bovine serum albumin (BSA)-coated substrates, and a similar asymmetry for PS and PE was observed. This result supported the notion that the asymmetry of PS and PE in SLBs was the result of generic hydrogen-bonding features adjacent to the lower lipid leaflet rather than specific binding sites. In other words, if lipids can hydrogen bond to a substrate more favorably than to water, then structural asymmetry can be induced.

These in vitro studies are highly reminiscent of the behavior of PE and PS in red blood cells where the same lipids partition into the inner leaflet adjacent to the cytoskeleton (Figure 1a).^8,23^ Supported bilayer partitioning behavior is remarkable because it suggests that H-bonding of two-dimensionally fluid lipids can play a significant role in a key structural component of bilayer membranes, namely, lipid asymmetry.

## Results

### Interleaflet Distribution of Tail-Labeled PC Lipids

In a first set of experiments, SLBs were formed on annealed planar glass supports from 100 nm diameter lipid vesicles containing 99.5 mol % POPC (1-palmitoyl-2-oleoyl-*sn*-glycero-3-phosphocholine) and 0.5 mol % NBD (7-nitro-2-1,3-benzoxadiazol-4-yl) -PC.^11^ The NBD fluorophore, attached to the acyl chain, enabled visualization and quantification of the labeled lipids by fluorescence microscopy (see Figure S1 for chemical structures).

To separate two leaflets, oxidized polydimethylsiloxane (PDMS) was gently brought into contact with the SLB within 10 min after bilayer formation. Notably, both the annealed glass coverslips and the PDMS used for unzipping were very smooth, with surface roughnesses confirmed by atomic force microscopy (AFM) (Figure S2). After dehydration, oxidized PDMS was peeled away from the SLBs, and lipid residues on the two substrates were viewed individually by fluorescence microscopy (Figure 2a). The dark horizontal stripe seen in the micrographs for both the upper and lower leaflets was introduced by running a tweezer tip over the SLB before unzipping was initiated. As can be seen, the width of the fluorescence line profile over the scratch was nearly identical in both leaflets (Figure 2b). Moreover, the fidelity of monolayer transfer was very high even from regions where the SLB was discontinuous. Together, these observations demonstrated that supported bilayers were decoupled into two monolayers, with the lower leaflet remaining on the glass and the upper leaflet being transferred to the oxidized PDMS (Figure 1d).

**Figure 2:**
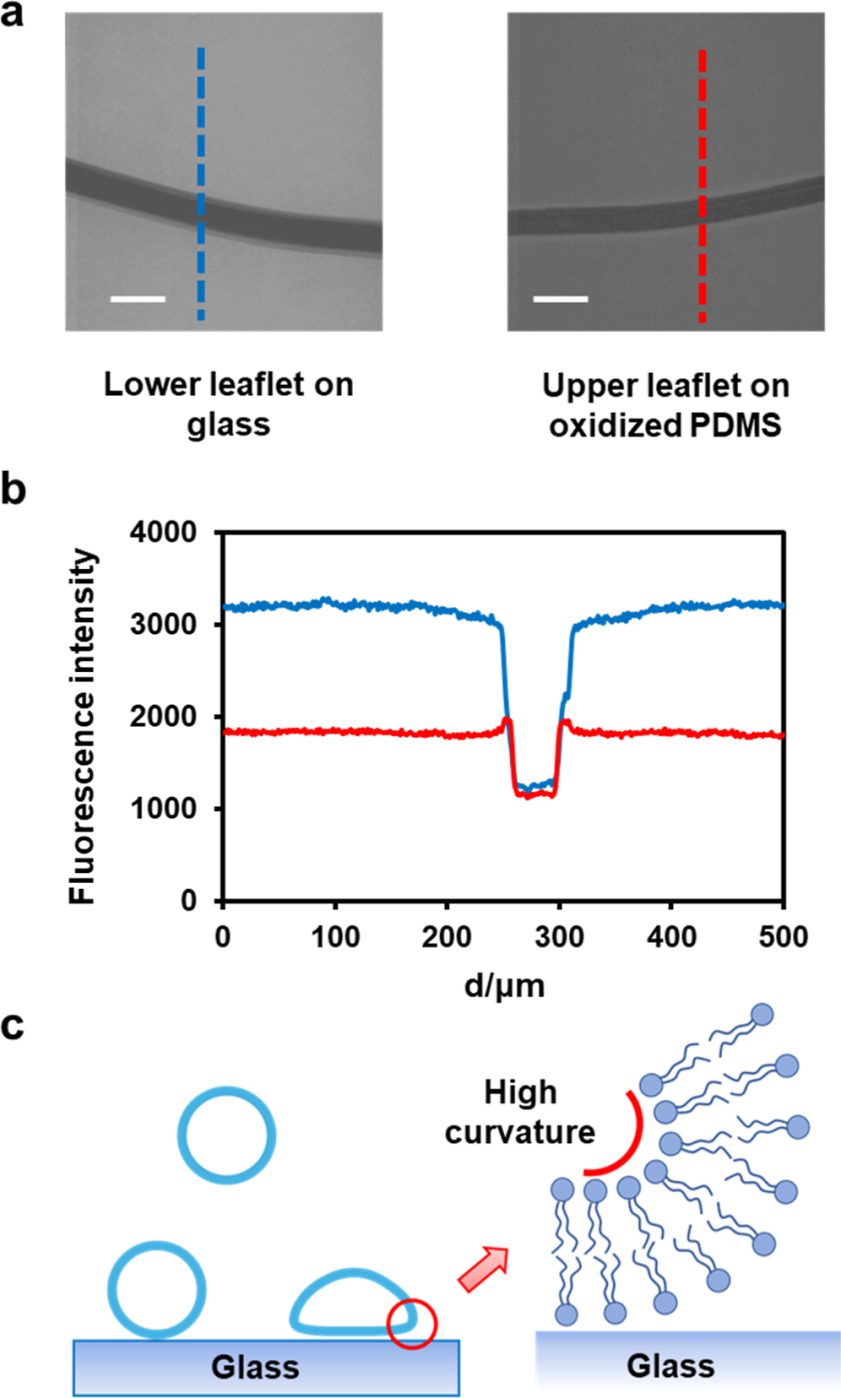
(a) Fluorescence images of decoupled monolayers from an SLB made from 99.5 mol % POPC and 0.5 mol % NBD-PC. The image contrast was set identically in both micrographs to directly reflect intensity differences. Scale bars: 100 μm. (b) Fluorescence intensity profiles from the dashed lines in (a). (c) Schematic illustration of the curvature-enhanced region during vesicle adsorption.

Furthermore, by applying the unzipping assay to freshly prepared asymmetric Langmuir–Blodgett (LB) bilayers,^24^ we could confirm that the unzipping process did not perturb the lipid distribution between the two leaflets (Figure S3). In fact, the bilayer became arrested as soon as conformal contact was made between the glass substrate and the oxidized PDMS slab (Figure S4).

For quantitative analysis of lipid asymmetry, the fluorescence intensity of NBD-PC on glass and PDMS was calibrated with LB monolayers (Figure S5). After calibration, the fluorescences from the two substrates could be directly compared. The results revealed that 66 ± 1% of the NBD-PC resided in the lower SLB leaflet, while 34 ± 1% resided in the upper one.

### Intrinsic Lipid Curvature Played a Role in Initial Asymmetry

The results from the lower leaflet measurement in Figure 2b are plotted as blue bars for NBD-PC in Figure 3a. The next question that was explored concerned whether the asymmetric NBD-PC distribution in the SLB was already present in the lipid vesicles or if it arose during vesicle fusion to the planar support. To explore this issue, a dithionite quenching assay^25^ was performed on 100 nm POPC vesicles containing 0.5 mol % NBD-PC (see Figure S6 for quenching assay details). The results showed that 60 ± 1% of the dye-labeled lipids were already present on the outer vesicle leaflet after extrusion (gray bar for NBD-PC in Figure 3a). As such, much of the initial lipid asymmetry in the SLBs can be attributed to the lipid distribution within the vesicles. Nevertheless, modest additional asymmetry resulted from the SLB formation.

**Figure 3:**
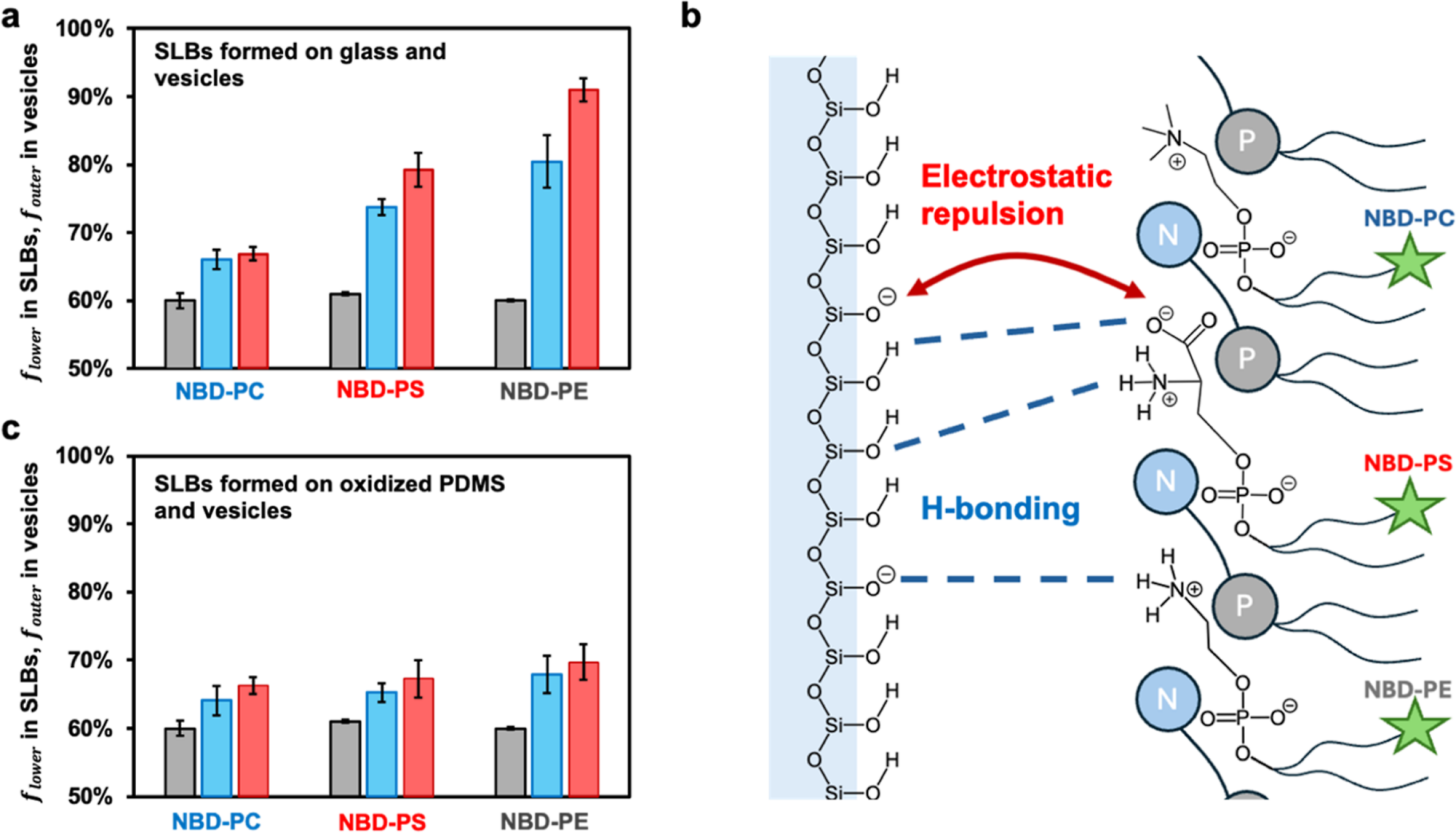
(a) Lower leaflet fractions of NBD-labeled lipids on glass supports and outer leaflet fractions from vesicles. Gray bars: outer leaflet fraction in vesicles; blue bars: lower leaflet fractions for SLBs formed in H_2_O buffer; red bars: lower leaflet fractions for SLBs formed in D_2_O buffer. Error bars represent standard deviations from five data sets. (b) Schematic illustration of interactions of PC, PE, and PS lipids with the glass surface. Note, the P–N dipole refers to the phosphate group (P) attached to the choline moiety (N) in the PC lipid headgroup. This is because experiments were performed in 99.5% POPC and 0.5% NBD-lipid. See the SI for a further discussion of glass chemistry. (c) Same experiments as in (a) but with oxidized PDMS substrates.

Intrinsic lipid curvature should have two opportunities to influence the initial NBD-PC distribution in the SLBs. First, there is an original distribution within the vesicles, which is well documented.^26^ Next, vesicle adsorption onto the glass substrate precedes SLB formation.^27^ When this happens, the vesicle shape on the glass support is distorted, which creates a ring of high curvature at the vesicle/substrate interface (Figure 2c).^28^ NBD-PC should be enriched in the outer leaflet in the distorted region by its intrinsic positive curvature but should be depleted from the inner leaflet in the same region. Since the lipids that form the SLB ultimately come from regions of the vesicle closer to the substrate, this should lead to an increase in the concentration of NBD-PC that ends up in the lower bilayer leaflet compared to the concentration in the upper leaflet. Indeed, this provides a plausible explanation for the enhancement from 60% NBD-PC in the outer vesicle leaflet to 66% found in the lower SLB leaflet after fusion.

The leaflet preference of NBD-PC within 100 nm vesicles may seem somewhat surprising, as one might expect a bulky dye on the end of a lipid acyl chain to preferentially partition into the inner vesicle leaflet, on the grounds that the dye-labeled lipid would have negative intrinsic curvature. However, previous literature indicates that the NBD moiety on NBD-PC tends to loop around so that it can reside closer to the headgroup/water interface.^29,30^ As such, the headgroup region ends up being bulkier, and NBD tail-labeled PC lipids possess positive intrinsic curvature. Such a positive curvature is consistent with the preferential partitioning of tail-labeled NBD-PC into the outer vesicle leaflet (Figure S7). To confirm this hypothesis, unzipping was performed with a more hydrophobic dye, tail-labeled TopFluor-PC.^31^ In this case, partitioning between the two SLB leaflets was nearly even (Figure S8).

Next, to probe whether vesicle size contributed to asymmetry, we repeated these measurements using vesicles of different diameters (50, 100, 200 nm; Figure S9). Generally, increasing vesicle size reduced both the asymmetry measured in vesicles and the magnitude of the initial SLB leaflet bias after fusion, consistent with weaker local curvature during adsorption and spreading. However, very small vesicles showed evidence for undergoing vesicle–vesicle fusion at the interface before fusing to the glass substrate, consistent with previous literature data (Figure S10).^28^

Finally, the role of H-bonding interactions between NBD-PC lipids and surface silanol groups on the glass support was investigated. To do this, SLBs were formed by vesicle fusion in D_2_O rather than H_2_O, as deuterium bonding is stronger.^32,33^ Unzipping experiments demonstrated that the NBD-PC distribution did not change within experimental error, with 34 ± 1% initially residing in the lower bilayer leaflet (red bar for NBD-PC in Figure 3a). This result was consistent with the idea that little, if any, H-bonding interactions occurred between PC headgroups and glass substrates. Indeed, the trimethylamine moiety on the PC headgroup cannot form H-bonds and the phosphate group may be too hindered by the choline moiety to accept H-bonds from surface silanol moieties (Figure 3b). This result also suggests that the NBD moiety does not significantly interact with the substrate via H-bond interactions.

### Hydrogen Bonding Promotes Asymmetry with NBD-PS and NBD-PE

The dithionite quenching experiments performed with 0.5 mol % NBD-PC were repeated with 0.5 mol % tail-labeled NBD-PS in 99.5 mol % POPC as well as with 0.5 mol % tail-labeled NBD-PE in 99.5 mol % POPC. The degree of asymmetry was essentially the same as found in 100 nm vesicles when using NBD-PC (gray bars for NBD-PS and NBD-PE in Figure 3a). Such a finding is consistent with the idea that NBD tail-labeled phospholipids loop around toward the headgroup/water interface, no matter whether it is PC, PS, or PE.

Next, lipid unzipping was performed with 0.5 mol % NBD-PS in POPC. The results showed significantly greater initial SLB asymmetry compared with NBD-PC (blue bar for NBD-PS in Figure 3a). In fact, 74 ± 1% NBD-PS resided in the lower bilayer leaflet when unzipping was performed within 10 min of vesicle fusion. Since the relative partitioning of NBD-PS and NBD-PC in 100 nm vesicles was almost the same, this result suggested that more substantial lipid reorganization occurred when vesicles containing PS headgroups were fused to planar glass supports compared with PC headgroups. Indeed, the amine moiety on PS should be capable of donating H-bonds to surface silanols, while the carboxylate moiety should be able to accept them (Figure 3b). Repeating this experiment in D_2_O confirmed the idea that H-bonding occurred in this case, as the initial fraction of NBD-PS in the lower leaflet rose to 79 ± 1% (red bar for NBD-PS in Figure 3a).

The NBD-PS results were striking as there should be a net electrostatic repulsion between the negatively charged PS lipids and the net negatively charged glass support, as approximately 25% of the surface silanols were deprotonated at pH 7.4.^34^ As such, H-bonding between PS headgroups and the support outweighed the repulsive electrostatic contribution to create SLBs that were initially about 3:1 enriched in dye-labeled PS lipids in the lower bilayer leaflet. Control experiments using the dithionate quenching assay demonstrated that D_2_O did not impact lipid asymmetry in the vesicles (Figure S11).

Vesicles containing 0.5 mol % NBD-PE in POPC showed an even greater propensity to form asymmetric SLBs than NBD-PS. In fact, 80 ± 4% of the NBD-PE initially partitioned into the lower bilayer leaflet (blue bar for NBD-PE in Figure 3a). Moreover, this number rose to 91 ± 2% when the experiment was repeated in D_2_O (red bar). These results suggested that the amine moiety on the NBD-PE headgroup was capable of hydrogen bonding with surface silanols (Figure 3c). Moreover, they were consistent with the idea that the carboxylate moiety on the PS lipids attenuated partitioning into the lower leaflet as opposed to enhancing it.

The positively charged amine moiety on NBD-PE should be able to donate an H-bond to either protonated or deprotonated surface silanol groups (Figure 3b). However, H-bonding between the amine and deprotonated silanol groups may be stronger than H-bonding with protonated silanols on electrostatic grounds.^35^ By contrast, the amine on NBD-PS should be subject to more H-bonding restrictions because the negatively charged carboxylate moiety should be electrostatically repelled from deprotonated silanols (Figure 3b). As such, even the amine on PS may need to bind in regions where there are fewer sites of deprotonation. This notion would be consistent with both greater initial SLB asymmetry for NBD-PE compared to that for NBD-PS as well as the more pronounced isotope effect.

To confirm the role of surface silanol groups on the initial SLB asymmetry in NBD-PS and NBD-PE, the planar glass substrate was replaced with an oxidized PDMS substrate, which had a substantially lower silanol density compared with an annealed glass support.^36,37^ SLBs readily formed on oxidized PDMS,^38,39^ and unzipping experiments analogous to those depicted in Figure 1d were performed. Results from experiments with NBD-PC, NBD-PS, and NBD-PE are listed in Figure 3c. Except for the use of a PDMS support, all conditions were otherwise identical with those used in Figure 3a. As can be seen, the initial SLB asymmetry for NBD-PC was almost the same within experimental error from the results obtained with a planar glass support (blue bar). By contrast, the asymmetry for NBD-PS and NBD-PE was only slightly greater than that for NBD-PC, and these differences were not outside the error bars of these measurements (blue bars).

The results described above indicate that the higher surface silanol density on glass was required to obtain the more pronounced asymmetry found with NBD-PS and NBD-PE. As expected, performing these experiments in D_2_O instead of H_2_O on oxidized PDMS led to substantially diminished isotope effects for PS and PE (red bars, Figure 3c) compared to those in Figure 3a, confirming the key role of H-bonding between headgroups and surface silanols in lipid asymmetry.

### Lipid Asymmetry Changes with Time

In the experiments described above, the SLBs were unzipped within 10 min of starting the vesicle fusion process. However, the lipid distribution at this time point may not represent the chemical equilibrium. To test this idea, unzipping was performed as a function of time up to 24 h after SLB formation. The results revealed that changes in the interleaflet distribution depended on the lipid headgroup chemistry (Figure 4a). For example, 66% of the NBD-PC was initially in the lower bilayer leaflet, but this fraction decreased over the next 24 h to exactly 50% (blue data points and line fit) as lipids diffused around the bilayer edge (Figure 4b).^13^ These data fit extremely well to a single exponential decay curve (eq 1)­

1 $$f_{lower}=a+be^{-t/\tau }$$

where *f*_lower_ represented the time-dependent lower leaflet fraction of the dye-labeled lipid, *a* stood for the lower leaflet fraction at equilibrium, while *b* was the difference between the initial fraction of the dye in the lower leaflet and the fraction at equilibrium. Next, *t* represents time in hours after SLB formation. Finally, the half-life for flipping, *t*_1/2_, could be calculated from τ by using eq 2

2 $$t_{1/2}=\ln2\times \tau$$

**Figure 4:**
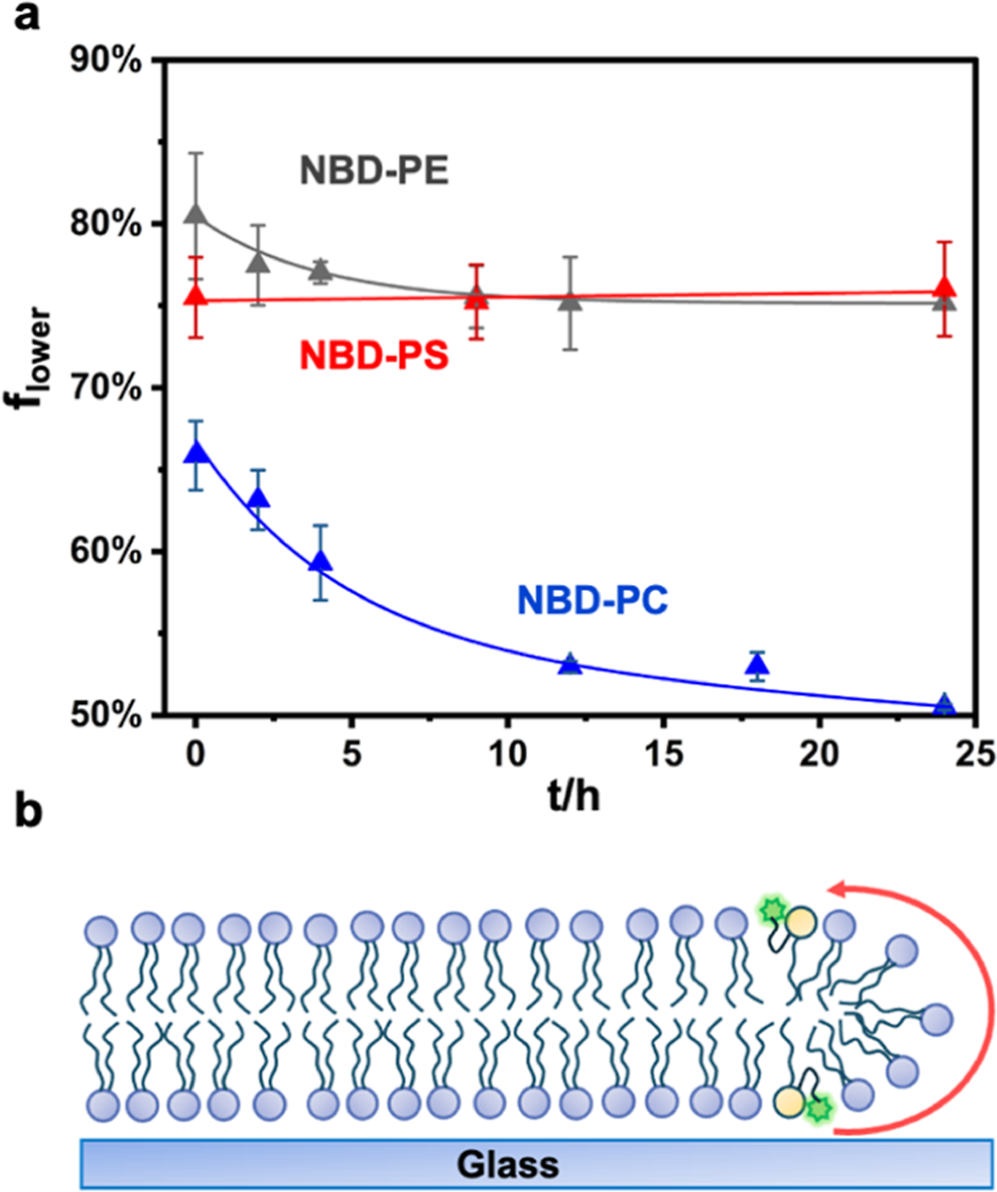
(a) Lower leaflet fraction of NBD-PC (blue), NBD-PS (red), and NBD-PE (gray) in SLBs as a function of time. Buffer: 20 mM HEPES with 50 mM NaCl at pH 7.4. (b) Schematic illustration of an NBD-labeled lipid flipping from the lower to upper leaflet via the membrane edge. See Supporting Information for additional discussion on flipping rates.

The fitted values using eq 2 are provided in Table 1.

**Table 1:** Flipping Kinetics and Equilibria of NBD-PC, NBD-PS, and NBD-PE in SLBs Containing 99.5 mol % POPC and 0.5 mol % NBD-Lipids in 20 mM HEPES and 50 mM NaCl Buffer at pH 7.4

lipids	NBD-PC	NBD-PS	NBD-PE
*t*_1/2_/h	5.1 ± 0.7	-	3.1 ± 0.8
*f*_lower (equilibrium)_	50 ± 1%	75 ± 1%	75 ± 1%

The half-time for flipping NBD-PC was 5.1 ± 0.7 h. The lipid distribution immediately after vesicle fusion was initially quite far from equilibrium but evolved exponentially toward it. This result represents a second piece of evidence that specific interactions between the NBD moiety and the glass surface should be minimal since NBD-PC had no preference for the lower leaflet once equilibrium was achieved.

The free energy difference for NBD-PC residing in the upper versus lower bilayer leaflet can be calculated using eq 3

3 $$\Delta G^{{}^{\circ}}=-RT\ln\left(\frac{f_{lower}}{f_{upper}}\right)$$

where Δ*G* ^0^ was the free energy difference between the two leaflets, *R* was the gas constant, and *T* = 294 K was the temperature. For NBD-PC, the ratio for [lower]/[upper] was equal to 1.0, and therefore Δ*G* ^0^ = 0. This makes sense, as the SLBs were planar, and the intrinsic curvature of NBD-PC ultimately should not matter at equilibrium.

Next, the gray data points and line fit represent the results for NBD-PE (Figure 4a). In this case, *t*_1/2_ was 3.1 ± 0.8 h. The fact that the half-life for flipping NBD-PE was only ∼60% of NBD-PC suggested a lower barrier for flipping PE. This result was curious as the ratio in the diffusion constant on glass between the two dye-labeled lipids was closer, 1.5 ± 0.1 μm^2^/s for NBD-PC and 1.8 ± 0.1 μm^2^/s for NBD-PE (Figure S12).

At equilibrium, the [lower]/[upper] leaflet ratio for NBD-PE was 3:1, which, according to eq 3, corresponded to a free energy preference for the lower leaflet, Δ*G* ^0^ = −2.7 kJ/mol. This value was approximately equal to the free energy of a fairly weak H-bond.

Finally, the partitioning of NBD-PS did not evolve as a function of time (red data points and curve in Figure 4a). There were likely offsetting factors that led to this result. First, NBD-PS lipids in vesicles should be expected to initially favor the lower leaflet on H-bonding and intrinsic curvature grounds, just like NBD-PE (Figure 2c). However, the net negative charge on NBD-PS should repel it from the substrate on electrostatic grounds. It would appear that these opposing factors essentially cancel each other out.

NBD-PS, like NBD-PE, partitioned at roughly a 3:1 ratio between the lower and the upper leaflets at equilibrium. As such, its free energy of interaction with the surface was also −2.7 kJ/mol. This result suggested that whatever favorable H-bonding interactions the carboxylate moiety might have had with the substrate was also roughly offset by electrostatic repulsion.

### The Influence of Buffer Conditions on Lipid Asymmetry

The data shown above demonstrated that both the lipid and substrate chemistry had a substantial influence on the asymmetry of lipids in SLBs. Of course, buffer conditions may also influence the lipid distribution. This should especially be true for NBD-PS, as electrostatic repulsion between the negatively charged PS headgroup and the anionic glass support should become screened from each other upon introducing sufficient salt into the buffer used in vesicle fusion. To test this hypothesis, unzipping experiments were run with NaCl concentrations ranging from 15 to 500 mM in a background of 20 mM HEPES buffer at pH 7.4. It should be noted that in the results described above, the concentration of NaCl was 50 mM, which gave rise to a lower leaflet NBD-PS fraction of 0.75 ± 0.01. Repeating this experiment with 15 mM NaCl caused the leaflet fraction to decrease to 0.69 ± 0.01. By contrast, this value rose to a peak of 0.76 ± 0.03 with 100 mM and 150 mM NaCl before falling to 0.73 ± 0.03 with 500 mM NaCl (Figure S13 shows the lower leaflet fraction data).

The data as a function of ionic strength were converted to $\ln\left(\frac{f_{lower}}{f_{upper}}\right)$ to make it proportional to an energy so that it could be fitted by a simple continuum electrostatic model consisting of an extended Debye–Hückel screening term plus a linear term (Figure 5 and eq 4)^40^

4 $$\ln\left[\frac{f_{lower}}{f_{upper}}\right]=\frac{A_{1}{\left[NaCl\right]}^{1/2}}{1+A_{2}{\left[NaCl\right]}^{1/2}}+B\left[NaCl\right]$$

where *A*_1_ and *A*_2_ were fitting constants associated with electrostatic screening, and *B* was a linear fitting constant representing the reduction in NBD-PS partitioning into the lower leaflet upon Na^+^ binding to either the carboxylate moiety on the NBD-PS, deprotonated surface silanols, or both (Figure 3b). The red line in Figure 5 shows the fit to the data.

**Figure 5:**
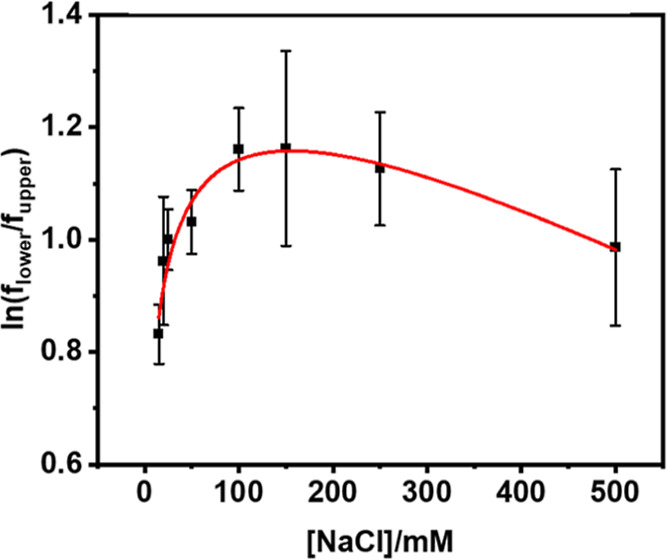
Initial asymmetry of NBD-PS in SLBs as a function of the NaCl concentration. Buffer: 20 mM HEPES with various NaCl concentrations at pH 7.4.

These results were consistent with the idea that electrostatic repulsion initially inhibited NBD-PS from more favorably partitioning into the lower bilayer leaflet in the absence of NaCl. As the salt concentration was increased, screening fostered a modest enhancement of PS in the lower leaflet before cation association with charged groups at higher salt concentrations caused this tendency to reverse.

What is striking about these results is the fact that the screening effect is quite small. Indeed, increasing the salt concentration from 15 mM to 150 mM only led to an enhancement in partitioning that was roughly equal to the deuterium isotope effect for NBD-PS (Figure 3a,c). This finding was consistent with the idea that hydrogen bonding was dominant over electrostatic repulsion under all ionic strengths that were tested. It should be noted that another possibility would be that the PS lipids in the lower leaflet may be partially protonated, as the hydronium ion concentration near the negatively charged surface should be higher than in the bulk solution.^41^ Curiously, however, previous experiments revealed little if any protonation of PS lipids in SLBs.^42^ Also, the ionic strength might have been lowered even further by removing the 20 mM HEPES buffer as well as 15 mM NaCl. Buffer removal, however, would create uncertainty in the protonation state of the amine and carboxylate moieties on the PS lipid. Moreover, the additional 15 mM NaCl was required to get the negatively charged vesicles containing NBD-PS to fuse to the surface.^11^

We also tested whether varying pH alters the initial leaflet distribution since pH changes both the degree of silanol deprotonation on glass and the protonation state of lipid headgroups. Unzipping experiments were therefore repeated immediately after bilayer formation at pH 5.0, 7.4, 8.5, and 9.0, while keeping the ionic strength fixed (Figure S14). As with the results for ionic strength, the pH experiments mostly revealed only modest changes in the lipid partition. At sufficiently high pH, however, deprotonation of the amine group on PE led to more pronounced changes.

### Relative Diffusion of NBD-Labeled Lipids on Glass vs PDMS

To examine lipid–substrate interactions, the diffusion of NBD-PC, NBD-PS, and NBD-PE was compared on glass and oxidized PDMS by fluorescence recovery after photobleaching (FRAP) assays (Figure S12).^43^ Figure 6 plots the diffusion ratios for oxidized PDMS vs glass, *D*_PDMS_/*D*_glass_. As can be seen, the diffusion for NBD-PC was only 8 ± 6% faster on PDMS compared with the glass coverslip. By contrast, the diffusion of NBD-PS increased by 89 ± 16% on PDMS, while it increased by 43 ± 3% for NBD-PE. Indeed, one would anticipate NBD-PS and NBD-PE to diffuse more slowly on glass relative to PDMS because of the higher density of surface silanols available for H-bonding on the glass surface. Of course, little if any change with NBD-PC should be expected because the choline moiety cannot form H-bonds with the surface silanols.

**Figure 6:**
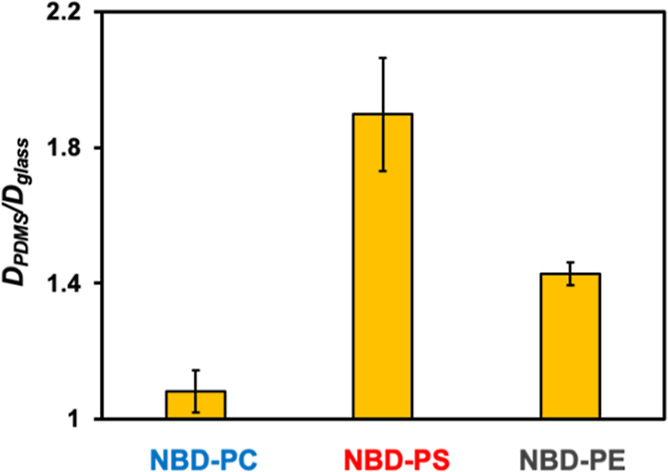
Diffusion coefficient ratio of NBD-labeled lipids in SLBs on oxidized PDMS vs glass supports. Buffer: 20 mM HEPES and 50 mM NaCl at pH 7.4.

The results in Figure 6 are consistent with greater H-bonding interactions between NBD-PS and the substrate compared with those in NBD-PE. Indeed, both the carboxylate and amine moieties from PS should have been able to interact with the substrate, while only the amine moiety on PE could do this. Moreover, the greater silanol density on glass may make it more likely that both the carboxylate and amine could be H-bonded simultaneously.

### Distribution of NBD-PC, NBD-PS, and NBD-PE on BSA-Coated Glass

In the experiments described above, supported bilayers were formed on glass and PDMS substrates. In a final set of experiments, we sought to perform the unzipping assay on BSA-coated substrates to explore lipid partitioning when the bilayer was adjacent to a protein layer. BSA was first deposited onto annealed glass, after which SLBs were formed on the BSA coating via vesicle fusion (Figure 7a).^44^ The bilayers were unzipped immediately following vesicle fusion using the same protocol applied to glass/PDMS-supported bilayers (Figure 7b) (see pages S5 and S6 in the Supporting Information section for details on the BSA coating and unzipping experiment). The results from unzipping performed within 10 min of bilayer formation are provided in Figure 7c.

**Figure 7:**
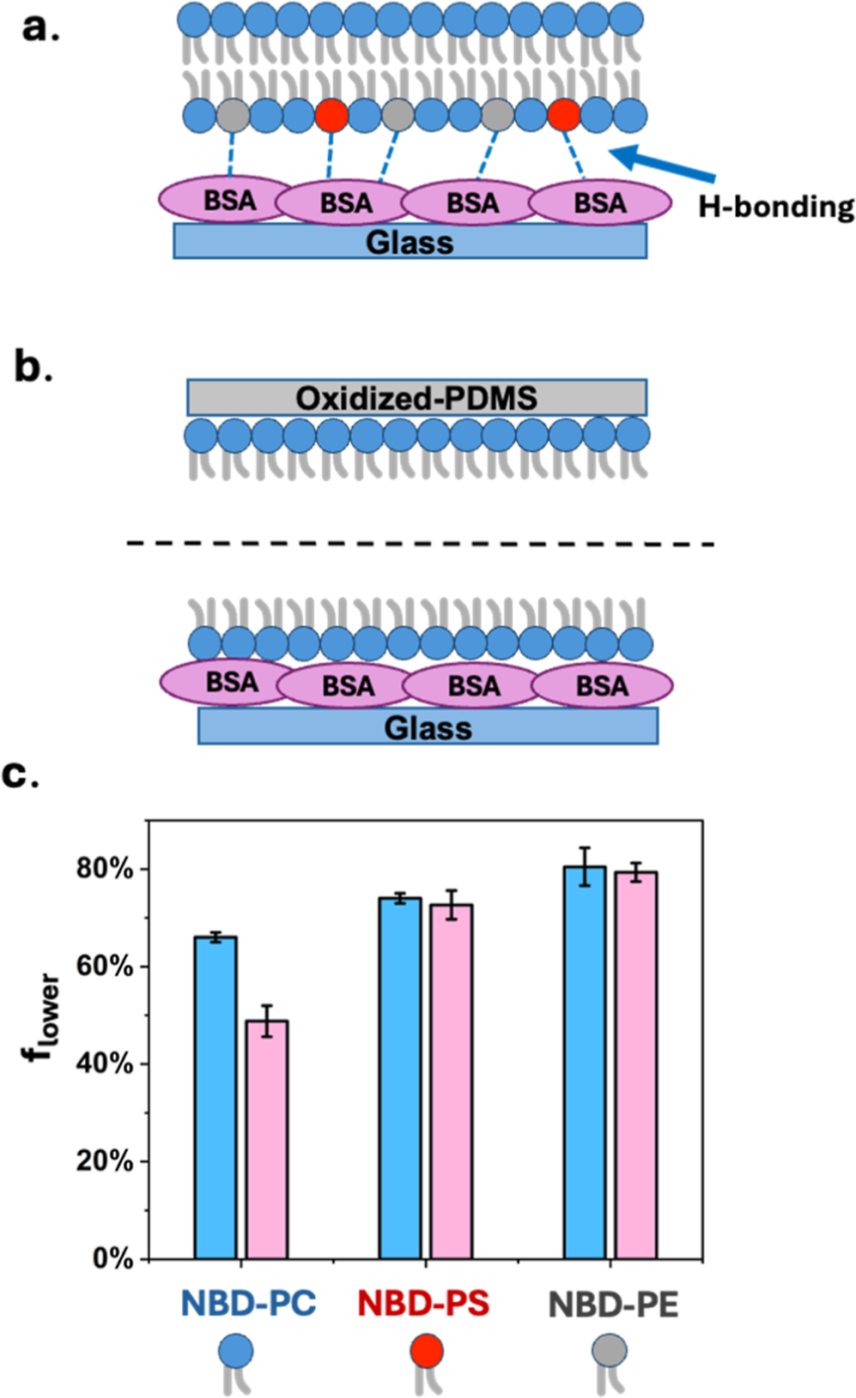
(a) Schematic of PS (red) and PE (gray) hydrogen bonding to BSA (pink)-coated glass. (b) Schematic of unzipping with BSA-coated glass. (c) Lower leaflet fractions of NBD-PC, NBD-PE, and NBD-PS on glass (blue bar) and on BSA-coated glass (pink bar).

As shown in Figure 7c, the asymmetry of NBD-PS and NBD-PE on the BSA-coated substrate (pink bars) was nearly identical with that initially observed on SLBs formed on glass (blue bars, identical to those in Figure 3a). This fact indicates that both the BSA-coated and glass substrates provided favorable H-bond acceptor sites, which partitioned PS and PE into the lower leaflets during bilayer formation. On the BSA-coated surface, the amide oxygen sites on the protein backbone should serve as H-bond acceptors. Like surface silanols on glass, the two-dimensional density of amide oxygens on proteins should be ∼5/nm^2^.^45^ As such, the free energy driving force for obtaining asymmetric lipid distributions with PS or PE stems from the fact that the amine moiety on the lipid headgroup forms modestly stronger H-bonds with the protein-coated substrate compared to amine–water and substrate–water H-bonds.

Strikingly, unlike PS or PE, the initial lower leaflet fraction of NBD-PC on the BSA-coated substrate was 49 ± 3% (pink bar), which was much different from the case for glass (blue bar in Figure 3a). This suggests that the initial mechanism of vesicle fusion on the protein-coated substrate should be different. For example, it may be the case that substrate-absorbed vesicles undergo vesicle–vesicle fusion before SLB formation can take place (Figure S10). This process would scramble and equilibrate the inner and outer leaflet lipid populations before the SLB formation occurred.

## Discussion and Conclusion

### Mechanisms for Supported Bilayer Asymmetry

Several previous studies have explored the lipid asymmetry in supported bilayers. For example, Parikh and co-workers investigated the role that electrostatics played in the asymmetric distribution of GM1 lipids in POPC membranes.^16^ GM1 is a negatively charged glycolipid, and the authors showed that it preferentially partitioned to the upper leaflet of the SLB to stay away from the negatively charged substrate. In this case, the GM1 distribution between the two bilayer leaflets is dominated by electrostatics because most of the H-bonding groups on GM1 lipids are alcohol moieties, which are too “hard” to favorably H-bond with surface silanol groups on the substrate surface.^46,47^

Another example of a lipid distribution that was dominated by electrostatics rather than H-bonding comes from the work of Bayerl and co-workers.^15^ In their paper, the authors showed that a positively charged lipid, containing a cationic quaternary nitrogen headgroup, preferred the lower leaflet, which should occur on electrostatic grounds. As was the case with GM1, the greasy cationic headgroup did not have favorable H-bonding sites.

The Parikh and Bayerl examples may be somewhat unusual, as it can be challenging to identify systems with sufficiently weak H-bonding such that electrostatics can be the dominant contributor to bilayer leaflet partitioning. However, Brisson and co-workers explored the partitioning behavior of dioleoylphosphatidylserine (DOPS) in dioleoylphosphatidylcholine (DOPC) membranes, which should lead to results dominated by H-bonding on both the silica and mica surfaces they used. The authors did not detect any asymmetric partitioning on silica, which may have been related to the design of their assay. By contrast, in the case of mica, the authors found that DOPS partitioned into the lower bilayer leaflet. They attributed this result to the presence of Ca^2+^ ions in their buffer. It should be noted that Ca^2+^ can bridge certain negatively charged oxyanions together, like phosphate, under some circumstances.^48^ However, there is no precedence for bridging carboxylate moieties with silanols by Ca^2+^. As such, it is more likely that DOPS partitioned to the lower leaflet through H-bonding interactions.

The previous literature makes it clear that H-bonding interactions between lipid headgroups and substrate surfaces have not been widely anticipated. Moreover, the literature often treated the idea of noncovalent interactions between functional groups that are electrostatically repulsive as surprising (e.g., PS lipids partitioning toward a negatively charged substrate or the favorable side-chain interactions between Arg residues).^49^ However, H-bonds, especially in aqueous systems, are controlled by a delicate balance of electrostatic, polarization, dispersion, and charge transfer interactions, among others. In the case of PE and PS lipids, favorable interactions with silanol and amide groups likely involve the role of charge transfer in the H-bond that is formed.^50^

### Biological Implications

Maintaining lipid asymmetry in cellular membranes is often presented as an energy-consuming process. If leaflet distributions were set and maintained solely by ATP-dependent transport (flippases and floppases), then the cost would be quite high. The results provided above, however, show that an H-bond-accepting interface can lead to a measurable thermodynamic preference. Indeed, on a planar glass support, we observe an equilibrium preference of ∼−2.7 kJ mol^–1^ for PE and PS for the lower, substrate-adjacent leaflet (Figure 4a). This thermodynamic preference is small on an absolute scale but sufficient to generate a 3:1 leaflet enrichment, supporting the idea that weak interfacial interactions can contribute to the biological structure.

PC behaves differently from PS and PE, showing no equilibrium preference for a glass or protein support (Figures 4a and 7). This result is qualitatively consistent with the comparatively more even distribution of PC between cell leaflet reports in red blood cells.^8^ The lack of preference suggests that the phosphate moiety is largely blocked from interacting with an adjacent substrate by choline groups on neighboring phosphatidylcholine lipids (Figure 3b). As such, it is only the end of the headgroups, beyond the phosphate moiety, that helps determine lipid partitioning preferences.

Finally, lipid–protein interactions do not require specific binding sites to induce lipid partitioning. Rather, a modest bias should arise from nonspecific, transient hydrogen bonding between PS/PE headgroups and cytoskeletal proteins based upon weakly favorable interactions between the amine moieties on lipid headgroups and the amide backbone. So long as hydrogen-bonding interaction sites are available on one side of the membrane (cytoskeletal side) and not the other (glycocalyx side), then asymmetric lipid structuring will occur (Figure 1a).

## Supplementary Material



## Notes

Materials and methods section; discussion of surface chemistry; lipid flipping comparison; chemical structure of lipids; surface characterization of glass and PDMS by AFM; experiments demonstrating no disturbance in SLB asymmetry from unzipping assays; restricted lateral mobility of lipids during unzipping; fluorescence calibration curves of lipid monolayer on glass and oxidized PDMS; dithionite quenching assay illustration; intrinsic curvature of NBD-lipid; symmetric TopFluor-PC distribution in SLBs; SLB asymmetry with varied vesicle size; illustration of two modes of vesicle rupture and fusion during SLB formation; vesicle asymmetry in H_2_O and D_2_O buffer conditions; lateral mobility of NBD-lipids on glass and oxidized PDMS; initial NBD-PS asymmetry with varied NaCl concentration; and impacts of buffer pH on lipid initial asymmetry (PDF)

## References

[1] Bretscher M. S. (1972). Asymmetrical
Lipid Bilayer Structure for Biological Membranes. Nature. New Biol..

[2] Bretscher M. S. (1973). Membrane
Structure: Some General Principles. Science.

[3] Doktorova M., Symons J. L., Levental I. (2020). Structural
and Functional Consequences
of Reversible Lipid Asymmetry in Living Membranes. Nat. Chem. Biol..

[4] Seigneuret M., Devaux P. F. (1984). ATP-Dependent Asymmetric Distribution
of Spin-Labeled
Phospholipids in the Erythrocyte Membrane: Relation to Shape Changes. Proc. Natl. Acad. Sci. U. S. A..

[5] Bassé F., Stout J. G., Sims P. J., Wiedmer T. (1996). Isolation
of an Erythrocyte
Membrane Protein That Mediates Ca^2+^-Dependent Transbilayer
Movement of Phospholipid. J. Biol. Chem..

[6] Purdon A. D., Rapoport S. I. (1998). Energy Requirements
for Two Aspects of Phospholipid
Metabolism in Mammalian Brain. Biochem. J..

[7] Williamson P., Antia R., Schlegel R. A. (1987). Maintenance
of Membrane Phospholipid
Asymmetry Lipid-Cytoskeletal Interactions or Lipid Pump?. FEBS Lett..

[8] Verkleij A. J., Zwaal R. F., Roelofsen B., Comfurius P., Kastelijn D., van Deenen L. L. (1973). The Asymmetric
Distribution of Phospholipids
in the Human Red Cell Membrane. A Combined Study Using Phospholipases
and Freeze-Etch Electron Microscopy. Biochim.
Biophys. Acta.

[9] Lorent J. H., Levental K. R., Ganesan L., Rivera-Longsworth G., Sezgin E., Doktorova M., Lyman E., Levental I. (2020). Plasma Membranes
Are Asymmetric in Lipid Unsaturation, Packing and Protein Shape. Nat. Chem. Biol..

[10] Schaut R. A., Lobello R. A., Mueller K. T., Pantano C. G. (2011). Characterization
of Boroaluminosilicate Glass Surface Structures by B *K*-Edge NEXAFS. J. Non-Cryst. Solids.

[11] Cremer P. S., Boxer S. G. (1999). Formation and Spreading
of Lipid Bilayers on Planar
Glass Supports. J. Phys. Chem. B.

[12] Arndt M., Alvadia C., Straub M. S., Clerico Mosina V., Paulino C., Dutzler R. (2022). Structural Basis for
the Activation
of the Lipid Scramblase TMEM16F. Nat. Commun..

[13] Marquardt D., Heberle F. A., Miti T., Eicher B., London E., Katsaras J., Pabst G. (2017). 1H NMR Shows
Slow Phospholipid Flip-Flop
in Gel and Fluid Bilayers. Langmuir.

[14] Sun S., Liu C., Rodriguez
Melendez D., Yang T., Cremer P. S. (2020). Immobilization
of Phosphatidylinositides Revealed by Bilayer Leaflet Decoupling. J. Am. Chem. Soc..

[15] Käsbauer M., Junglas M., Bayerl T. M. (1999). Effect of Cationic Lipids in the
Formation of Asymmetries in Supported Bilayers. Biophys. J..

[16] Shreve A. P., Howland M. C., Sapuri-Butti A. R., Allen T. W., Parikh A. N. (2008). Evidence
for Leaflet-Dependent Redistribution of Charged Molecules in Fluid
Supported Phospholipid Bilayers. Langmuir.

[17] Stanglmaier S., Hertrich S., Fritz K., Moulin J.-F., Haese-Seiller M., Rädler J. O., Nickel B. (2012). Asymmetric Distribution of Anionic
Phospholipids in Supported Lipid Bilayers. Langmuir.

[18] Fadeel B., Xue D. (2009). The Ins and Outs of
Phospholipid Asymmetry in the Plasma Membrane:
Roles in Health and Disease. Crit. Rev. Biochem.
Mol. Biol..

[19] Crane J. M., Kiessling V., Tamm L. K. (2005). Measuring Lipid Asymmetry in Planar
Supported Bilayers by Fluorescence Interference Contrast Microscopy. Langmuir.

[20] Jönsson P., Beech J. P., Tegenfeldt J. O., Höök F. (2009). Shear-Driven
Motion of Supported Lipid Bilayers in Microfluidic Channels. J. Am. Chem. Soc..

[21] Gerelli Y., Porcar L., Lombardi L., Fragneto G. (2013). Lipid Exchange and
Flip-Flop in Solid Supported Bilayers. Langmuir.

[22] Karedla N., Schneider F., Enderlein J., Chen T. (2025). Leaflet-Specific Structure
and Dynamics of Solid and Polymer Supported Lipid Bilayers. Angew. Chem. Int. Ed..

[23] Williamson P., Bateman J., Kozarsky K., Mattocks K., Hermanowicz N., Choe H.-R., Schlegel R. A. (1982). Involvement of Spectrin in the Maintenance
of Phase-State Asymmetry in the Erythrocyte Membrane. Cell.

[24] Kurniawan J., Ventrici de Souza J. F., Dang A. T., Liu G., Kuhl T. L. (2018). Preparation
and Characterization of Solid-Supported Lipid Bilayers Formed by Langmuir–Blodgett
Deposition: A Tutorial. Langmuir.

[25] Angeletti C., Nichols J. W. (1998). Dithionite Quenching
Rate Measurement of the Inside–Outside
Membrane Bilayer Distribution of 7-Nitrobenz-2-Oxa-1,3-Diazol-4-Yl-Labeled
Phospholipids. Biochemistry.

[26] Kamal M. M., Mills D., Grzybek M., Howard J. (2009). Measurement of the
Membrane Curvature Preference of Phospholipids Reveals Only Weak Coupling
between Lipid Shape and Leaflet Curvature. Proc.
Natl. Acad. Sci. U. S. A..

[27] Johnson J. M., Ha T., Chu S., Boxer S. G. (2002). Early Steps of Supported Bilayer
Formation Probed by Single Vesicle Fluorescence Assays. Biophys. J..

[28] Schönherr H., Johnson J. M., Lenz P., Frank C. W., Boxer S. G. (2004). Vesicle
Adsorption and Lipid Bilayer Formation on Glass Studied by Atomic
Force Microscopy. Langmuir.

[29] Huster D., Müller P., Arnold K., Herrmann A. (2001). Dynamics of Membrane
Penetration of the Fluorescent 7-Nitrobenz-2-oxa-1,3-diazol-4-yl (NBD)
Group Attached to an Acyl Chain of Phosphatidylcholine. Biophys. J..

[30] Loura L. M. S., Ramalho J. P. P. (2007). Location and Dynamics of Acyl Chain
NBD-Labeled Phosphatidylcholine (NBD-PC) in DPPC Bilayers. A Molecular
Dynamics and Time-Resolved Fluorescence Anisotropy Study. Biochim. Biophys. Acta BBA—Biomembr..

[31] Regan D., Williams J., Borri P., Langbein W. (2019). Lipid Bilayer Thickness
Measured by Quantitative DIC Reveals Phase Transitions and Effects
of Substrate Hydrophilicity. Langmuir.

[32] Némethy G., Scheraga H. A. (1964). Structure of Water
and Hydrophobic Bonding in Proteins.
IV. The Thermodynamic Properties of Liquid Deuterium Oxide. J. Chem. Phys..

[33] da
Cruz Garcia M. L., Paixão R. R., Pazin W. M., Oliveira O. N., Cremer P. S., Carlos R. M. (2024). Binding of Cis-[Ru­(Phen)_2_(3,4Apy)_2_]^2+^ to Model Lipid Membranes:
Implications for New Tools in the Development of Antiamyloid Drugs. Langmuir.

[34] Ong S., Zhao X., Eisenthal K. B. (1992). Polarization of Water Molecules at
a Charged Interface: Second Harmonic Studies of the Silica/Water Interface. Chem. Phys. Lett..

[35] Rimola A., Sodupe M., Ugliengo P. (2009). Affinity Scale for
the Interaction
of Amino Acids with Silica Surfaces. J. Phys.
Chem. C.

[36] Schrader A.
M., Monroe J. I., Sheil R., Dobbs H. A., Keller T. J., Li Y., Jain S., Shell M. S., Israelachvili J. N., Han S. (2018). Surface Chemical Heterogeneity Modulates Silica Surface Hydration. Proc. Natl. Acad. Sci. U. S. A..

[37] Hillborg H., Ankner J. F., Gedde U. W., Smith G. D., Yasuda H. K., Wikström K. (2000). Crosslinked Polydimethylsiloxane
Exposed to Oxygen
Plasma Studied by Neutron Reflectometry and Other Surface Specific
Techniques. Polymer.

[38] Hovis J. S., Boxer S. G. (2001). Patterning and Composition
Arrays of Supported Lipid
Bilayers by Microcontact Printing. Langmuir.

[39] Goodchild J. A., Walsh D. L., Laurent H., Connell S. D. (2023). PDMS as a Substrate
for Lipid Bilayers. Langmuir.

[40] Samson E., Lemaire G., Marchand J., Beaudoin J. J. (1999). Modeling Chemical
Activity Effects in Strong Ionic Solutions. Comput. Mater. Sci..

[41] Jung H., Robison A. D., Cremer P. S. (2009). Detecting Protein–Ligand
Binding
on Supported Bilayers by Local pH Modulation. J. Am. Chem. Soc..

[42] Poyton M. F., Cremer P. S. (2013). Electrophoretic Measurements of Lipid Charges in Supported
Bilayers. Anal. Chem..

[43] Soumpasis D. M. (1983). Theoretical
Analysis of Fluorescence Photobleaching Recovery Experiments. Biophys. J..

[44] Diaz A. J., Albertorio F., Daniel S., Cremer P. S. (2008). Double Cushions
Preserve Transmembrane Protein Mobility in Supported Bilayer Systems. Langmuir.

[45] Graham D. E., Phillips M. C. (1979). Proteins at Liquid Interfaces. J. Colloid Interface Sci..

[46] Flór M., Vorobev V., Mandalaparthy V., Van Der Vegt N. F. A., Cremer P. S., Roke S. (2025). Unraveling the Molecular
Pathways
for Structure “Making” and “Breaking”
by Ions in Water. J. Am. Chem. Soc..

[47] Pearson R. G. (1963). Hard and
Soft Acids and Bases. J. Am. Chem. Soc..

[48] Bilkova E., Pleskot R., Rissanen S., Sun S., Czogalla A., Cwiklik L., Róg T., Vattulainen I., Cremer P. S., Jungwirth P., Coskun Ü. (2017). Calcium Directly
Regulates Phosphatidylinositol-4,5-Bisphosphate Headgroup Conformation
and Recognition. J. Am. Chem. Soc..

[49] Vazdar M., Heyda J., Mason P. E., Tesei G., Allolio C., Lund M., Jungwirth P. (2018). Arginine “Magic”:
Guanidinium
Like-Charge Ion Pairing from Aqueous Salts to Cell Penetrating Peptides. Acc. Chem. Res..

[50] Reed A. E., Weinhold F., Curtiss L. A., Pochatko D. J. (1986). Natural
Bond Orbital
Analysis of Molecular Interactions: Theoretical Studies of Binary
Complexes of HF, H_2_O, NH_3_, N_2_, O_2_, F_2_, CO, and CO_2_ with HF, H_2_O, and NH_3_. J. Chem. Phys..

