# Supplementary material for: Lipid–Substrate Interactions Lead to Bilayer Asymmetry
Source: J Am Chem Soc. 2026 May 12;148(20):20898–907. doi: 10.1021/jacs.6c04860 (PMC13220268; doi:10.1021/jacs.6c04860)
Supplement: Supplementary file 1 [file ja6c04860_si_001.pdf]

Supporting Materials for "Lipid-substrate Interactions Lead to Bilayer Asymmetry"

Ruofei Wang,[1] Ella Gregory,[1] Brandon A. Oswald,[1] Tinglu Yang,[1] Paul S. Cremer[1,2*]

[1]Department of Chemistry, Penn State University, University Park, Pennsylvania 16802, United States

[2]Department of Biochemistry and Molecular Biology, Penn State University, Pennsylvania 16802, United States

*Corresponding Author Email Address: psc11@psu.edu

Materials and Methods:

1-Palmitoyl-2-oleoyl-*sn*-glycero-3-phosphocholine (POPC), 1-palmitoyl-2-oleoyl-sn-glycero-3-phospho-L-serine (sodium salt) (POPS), 1-palmitoyl-2-{12-[(7-nitro-2-1,3-benzoxadiazol-4-yl)amino]dodecanoyl}-sn-glycero-3-phosphocholine (16:0-12:0 NBD-PC), 1-palmitoyl-2-{12-[(7-nitro-2-1,3-benzoxadiazol-4-yl)amino]dodecanoyl}-sn-glycero-3-phosphoserine (ammonium salt) (16:0-12:0 NBD-PS), 1-palmitoyl-2-{12-[(7-nitro-2-1,3-benzoxadiazol-4-yl)amino]dodecanoyl}-sn-glycero-3-phosphoethanolamine (16:0-12:0 NBD-PE) and 1-oleoyl-2-(6-((4,4-difluoro-1,3-dimethyl-5-(4-methoxyphenyl)-4-bora-3a,4a-diaza-s-indacene-2-propionyl)amino)hexanoyl)-sn-glycero-3-phosphocholine (TopFluor-PC) were purchased from Avanti Polar Lipids (Alabaster, AL). Texas Red-1,2-dihexadecanoyl-*sn*-glycero-3-phosphoethanolamine, triethylammonium salt (TR-DHPE) was purchased from Life Technologies (Grand Island, NY). 4-(2-hydroxyethyl)piperazine-1-ethanesulfonic acid (HEPES, >99.5%) was purchased from Sigma-Aldrich (St. Louis, MO). Sodium chloride (NaCl, >99.9%) was purchased from DOT Scientific Inc. (Burton, MI). Bovine serum albumin (BSA) was obtained from VWR Life Sciences (Solon, OH). Purified water was obtained from a Nanopure Ultrapure Water System (18.2 MΩ·cm, Barnstead, Dubuque, IA). Glass coverslips (24 × 40 mm and 22 x 22 mm, No. 1.5) were purchased from Fisher Scientific (Waltham, MA). PDMS (polydimethylsiloxane, Dow Corning Sylgard Silicone Elastomer-184) was obtained from Krayden, Inc. (El Paso, TX).

Small Unilamellar Vesicle (SUV) Preparation

Phospholipids were mixed in chloroform and then dried by purging with nitrogen gas. The mixtures contained varying mole fractions of POPC and NBD-labeled lipids which were desiccated under vacuum for three hours before reconstituting in 20 mM HEPES buffer with certain amount of NaCl (generally 50 mM) to a concentration of 0.5 mg/mL. After the lipids were fully dissolved in buffer solution, the solutions were subjected to ten freeze-thaw cycles by successive immersion in liquid nitrogen followed by warm water (~40 °C). This was followed by fifteen extrusion cycles using a Lipex extruder (Northern Lipids Inc., Vancouver, Canada) through polycarbonate filters (Whatman) with 100 nm diameter holes. The size of the extruded vesicles was determined by dynamic light scattering (DLS) (Brookhaven Instruments 90Plus Particle Size Analyzer), and the mean diameter was around 120 nm. Vesicle solutions were stored at 4 ºC until use. For experiments at different pH values, vesicles were prepared following the same procedure, whereby the only difference is that 70 mM NaCl was introduced into 18.2 MΩ·cm water solution was added instead of buffer. The 70 mM NaCl solution as adjusted to pH 5.0, 7.4, 8.5, and 9.0 using dropwise addition of NaOH/HCl.

For experiments with other vesicle diameters, vesicles were extruded through membranes with pore sizes of 50 nm and 200 nm, respectively. Dynamic light scattering measurements yielded mean diameters of approximately 80 nm and 190 nm for these two vesicle populations.

Glass Cleaning Procedure

22×40 mm glass coverslips (thickness of #1½) were used for supporting fluid lipid bilayers (Corning Inc., Corning, NY). The coverslips were prepared by first boiling them in a 1:6 solution of 7X cleaning solution (MP Biomedicals, Solon, OH) for at least 2 hours. This was followed by rinsing with copious amounts of purified water before drying thoroughly with nitrogen gas. The

cover slips were then annealed for six hours at 550 °C. The cleaned and annealed coverslips were stored in boxes until use.

Supported Lipid Bilayer (SLB) Formation

Supported bilayers were formed via the vesicle fusion method.[1] A 50 µL suspension of SUVs (0.5 mg/mL, 100 nm diameter) was added to a PDMS well with an open bottom situated on either a glass coverslip or an oxidized PDMS substrate. The sample was incubated for 10 minutes to allow vesicle rupture and bilayer formation to occur. Excess and unruptured vesicles were then thoroughly washed away with buffer.

Dithionite Quenching Assay

Fluorescence quenching measurements were performed on an SLM 8000C spectrofluorometer (excitation 475 nm; emission 530 nm). The digital fluorescence output from the fluorometer was recorded at 0.5 Hz. Vesicles were suspended in 1 mL of 20 mM HEPES buffer at 25 °C (final phospholipid concentration of 0.5 mg/ml). 25 µL of 1 M dithionite (pH 10) was added to the cuvette, which was used as the t = 0 quenching time for a given measurement.

Fluorescence Recovery after Photobleaching (FRAP) Measurements

After forming an SLB as described above, bilayer material was removed in long stripes by scratching the surface of the glass coverslip with a pair of tweezers.[2] This was done to provide contrast between dark scratched regions and bright fluorescent regions under a fluorescence microscope. Such scratches made it easier to focus the microscope to the plane of the bilayer surface and keep track of specific locations on the surface. After scratching, the bilayer was rinsed copiously with buffer before imaging.

FRAP measurements were carried out with a Nikon Eclipse TE2000 U inverted microscope through a Nikon 10x Plan Apo air objective (N.A. = 0.45) with a Nikon LU-N3 laser unit. The circular region of interest (ROI) with a radius of ω (where ω was approximately 10 µm) was bleached by high intensity laser scanning controlled by a Bruker galvanometer mini-scanner. For NBD-labeled lipids, we chose 488 nm laser for photobleaching, with a 50 µs dwell time. After photobleaching, the bleached components diffused away and new fluorophores could diffuse in, leading to fluorescence recovery. To track this process, a time-dependent imaging series after photobleaching was acquired with NIS-Elements: (1) every second for the first 10 seconds, (2) every 3 seconds starting at 10 seconds for a minute, (3) every 20 seconds starting at the minute mark for 20 minutes, (3) every one minute starting at 20 minutes for one hour. If needed, additional points were measured (4) every five minutes starting from the 1-hour mark for the rest of the recovery.

To determine the diffusion coefficient,[3] the relative fluorescence intensity within the ROI was calculated with three adjacent regions as references. The fluorescence of the bleached spot was normalized to three unbleached reference spots, and the recovery fraction (y-axis) was plotted over time (x-axis) as:

$$F(t) = \frac{F_t - F_0}{1 - F_0} \quad \text{(Eqn. S1)}$$

where F_t is the normalized fluorescence at time t and F_0 is the normalized fluorescence at t = 0. The recovery fraction was calculated using Eqn. S2, which was used for fitting:

$$f(t) = \frac{F(t) - F(0)}{1 - F(0)} \quad \text{(Eqn. S2)}$$

$f(t)$ could be explicitly fit by modified Bessel functions, as shown in Eqn. S3:

$$f(t) = A \cdot \exp\left(-\frac{2\tau_D}{t}\right)\left[\mathbf{I_0}\left(\frac{2\tau_D}{t}\right) + \mathbf{I_1}\left(\frac{2\tau_D}{t}\right)\right] + b \quad \text{(Eqn. S3)}$$

where $\mathbf{I_0}$ and $\mathbf{I_1}$ stood for modified Bessel functions of first kind. Value of τ was used to calculate the diffusion constant *D* with the following equation:

$$D = \frac{w^2}{4\tau} \quad \text{(Eqn. S4)}$$

Mobile Fraction, M, of SLBs can be assessed by the equation below.

$$M = A + b \quad \text{(Eqn. S5)}$$

Bilayer Unzipping Assays

I. PDMS cleaning and oxidation.

PDMS was mixed at a 10:1 mass ratio of base to cross-linker and degassed under vacuum for 1 hour.[4] The resulting PDMS gel solution was poured onto a silicon wafer to a thickness of ~3 mm and placed in a 70 °C oven for twelve hours. Prior to use, the PDMS was wrapped with Scotch tape, cut into 5 mm × 5 mm pieces, and rinsed with ethanol in a cleanroom environment to remove any adhesive residue from the tape. The cleaned PDMS was then dried using a flow of N_2 gas.

To prepare the PDMS for further use, it was oxidized using an oxygen plasma cleaner. The plasma chamber was flushed with oxygen three times, ensuring the pressure went down to 0.3 Torr. The plasma was activated once the pressure stabilized at 0.55 Torr. The PDMS pieces were oxidized for 60 seconds and immediately immersed in water after oxidation.

II. "Sandwich" formation under water

Supported lipid bilayers were formed using the assay described above. SLBs were first formed inside open ring-shaped PDMS wells on 22 x 40 mm glass coverslips. The diameter of the well was 0.80 cm ± 0.05 cm, and the rim of the wells was 0.20 cm ± 0.05 cm. The PDMS rings were first cleaned and then attached to the glass. Marks were made on the opposite side of the glass slides to denote the region where the bilayer was formed. Each PDMS well on glass could hold up to ~50

μL solution. Vesicle solutions were injected into the wells, and incubated for 10 minutes, before rinsing copiously with buffer solution. Thin lines were scratched into the bilayer with a pair of tweezers,[2] so as to ensure enough contrast under the fluorescence microscope. Moreover, the scratches acted as spatial markers for lipid transfer to oxidized PDMS.

To perform an assay, a glass coverslip with an SLB was immersed in a petri dish that was filled with degassed 18.2 MΩ·cm water. Next, the PDMS ring was gently peeled away with a pair of tweezers under water. The oxidized PDMS was then brought into contact with the glass coverslip carrying the SLB while still under water. Caution needed to be taken to avoid any trapped dust particles or air bubbles getting lodged between the two substrates. As such, this procedure was conducted slowly and inside a class 1000 clean room. The sandwich with the SLB inside was removed from bulk water with a pair of tweezers and immediately transferred into a petri dish inside a desiccator. All subsequent steps were conducted in an ambient environment with less than 40% relative humidity. The desiccator was linked to a vacuum pump which produced a 10 mTorr vacuum. The glass sandwich was left under vacuum for 2 hours, allowing the water to evaporate. After taking the glass sandwich out of the desiccator, a razor blade was used to separate the sandwich.

As soon as the PDMS contacted the bilayer on the glass substrate, the bulk water was displaced, and the lateral mobility of the lipids was arrested. Significantly, this stopped lipid flip-flip from occurring (Fig. S4). Therefore, the asymmetry state of the bilayer was preserved at exactly the time point when conformal contact was made between the PDMS slab and the glass support.

III. Lipid asymmetry quantification

After the lipid bilayer unzipping assay was performed, the lower bilayer leaflet on glass and the upper bilayer leaflet on oxidized PDMS were imaged separately on a Nikon Eclipse TE2000 U inverted microscope equipped with a 10X Nikon Plan Apo air objective (N.A. = 0.45) and a 555 nm light source. Imaging was performed as quickly as possible to minimize photobleaching caused by both excitation and ambient light. The oxidized PDMS slab (~3 mm thick) was substantially thicker than a #1.5 glass coverslip (~0.15 mm). To minimize optical distortion due to the oxidized PDMS slab thickness, both monolayers were oriented facing toward the objective lens during imaging. For each sample, 3 paired images were acquired from distinct regions on both substrates. In each image, the average fluorescence intensity was determined using 3 regions of interest (ROIs) of 5000 μm² each. The mean value of these 3 × 3 measurements was employed to quantify lipid asymmetry from an individual sample.

BSA coating and Unzipping

BSA was prepared at 1 mg/mL in 20 mM HEPES buffer (pH 7.40) containing 50 mM NaCl. Before use, the solution was diluted to 0.01 mg/mL and centrifuged at 10,000 rpm for 10 minutes to remove aggregates. To coat the glass surface, the 0.01 mg/mL BSA solution was added to a microwell on the glass support and incubated for 40 minutes.

After a 40-minute incubation period, the BSA-coated glass was rinsed with high-ionic-strength HEPES buffer (pH 7.40, 300mM NaCl) to remove excess protein. Prior to vesicle addition, approximately 40 µL of residual buffer remained in the PDMS well. Next, 50 µL of vesicle suspension (99.5% POPC, 0.5% NBD-labelled dye in 20 mM HEPES and 50 mM NaCl at pH 7.4) was introduced into the well and incubated for 20 minutes.[5] The supported lipid bilayers (SLBs) were subsequently rinsed with DI water before the unzipping step. The unzipping followed exactly the same procedure as described above.

Atomic Force Microscopy Measurements

Surface morphology and topographical roughness were characterized using a NanoScope IIIa MultiMode AFM (Digital Instruments). All data acquisition and post-processing, including root-mean-square (RMS) roughness (Rq) calculations, were performed using NanoScope 5.30r3sr3 software. Images were acquired at a resolution of 512 × 512 pixels. Annealed glass coverslips and polydimethylsiloxane (PDMS) surfaces were characterized in tapping mode under ambient conditions. NSC15 silicon cantilevers (µmash) were employed, featuring a nominal resonance frequency of 325 kHz and a spring constant of 46 N/m. To ensure statistical reproducibility, roughness values were averaged over at least three independent scan areas (1 x 1 µm) per sample. Raw topographical data were subjected to a first order flattening routine to remove longitudinal tilt and scanner bow artifacts prior to analysis.

Discussion about Surface Chemistry in our System

I. Confined Water

Techniques such as sum frequency vibrational spectroscopy (SFVS), atomic force microscopy (AFM), and neutron reflectivity confirm that there is "confined" water between the lower leaflet of a supported bilayer and the glass substrate.[6] This water layer is typically measured to be between 0.5 nm and 1.0 nm thick, which represents 2 to 4 water layers. Such water acts to attenuate hydrogen bonding between the lipid head groups and the substrate silanol moieties. This is the case because water-headgroup and water-silanol interactions compete with lipid headgroup – silanol interactions.

However, not all types of hydrogen bonding have the same free energy. In the present system, amine – silanol interactions displace some of the interactions between the water and the substrate as well as those between water and the lipid head groups (and also form new water-water H-bonds). This competition is not merely statistical. Rather, the formation of lipid head group – silanol interactions is free energy favorable by about 2.7 kJ/mol as shown by the calculation associated with Eqn. 3 on p. 5 of the manuscript.

II. Surface Chemistry of Borosilicate Glass

The surface properties of borofloat glass have been well characterized in previous studies. In many respects the surface is similar to pure silica, as it is typically dominated by surface silanols, but there are a few differences. Firstly, at neutral pH, there are approximately 5 silanol groups per square nanometer.[7] Moreover, under water the terminal silanol groups become partially deprotonated to yield a net negative surface charge. The surface charge density at pH 7 typically ranges from −0.2 to −0.6 mC/m^{-2}, depending on the ionic strength.[8] Note that the degree of deprotonation rises as the ionic strength increases.

Next, the leaching rate of Na^+ has been extensively studied. Near neutral pH between 20 °C and 25 °C, leaching rates are found to be between 0.02 and 0.05 μg/ cm^2 per day.[9] This rate can be slightly sped up under basic conditions. It should be noted that most of our studies were conducted in 20 mM HEPES at pH 7.4 with an additional 50 mM NaCl in solution. As such, the ionic strength of our buffer overwhelms the very tiny quantities of Na^+ that can leach out of the glass.

The interaction between Na^+ and deprotonated surface silanols is weak. The electrostatics mostly follow the Gouy-Chapman model, that is consistent with a simple electrostatic double layer model.[10]

We have previously reported that our glass preparation procedures yield a root-mean-square (RMS) roughness of 0.13 nm over a 1 μm^2 area.[11] Since the bilayer is ~4.5 nm high, this is quite flat. We are able to achieve this degree of smoothness by annealing the borofloat glass in a kiln for 6 hours at 550 °C, which is just below the glass transition temperature of 560 °C. AFM measurements were repeated for the borofloat glass used in this experiment, which yielded comparable RMS values (0.16 ± 0.02 nm over 1 μm^2 area). (Fig. S2)

III. Types of Surface Silanols

It is assumed that vicinal silanols are the easiest to deprotonate because H-bonding by the deprotonated site with the neighboring OH group stabilizes proton removal.[7] By contrast, both isolated and geminal silanol groups are more difficult to deprotonate and typically have pK_a values at least a full pH unit above 7.4, where most of the work in this manuscript was done.

Vicinal sites on borofloat glass are known to be patchy,[12] so a fraction of the deprotonated sites may be clustered rather than uniformly distributed. As such, an isolated H-bond donating lipid (i.e., 0.5 mol% of NBD-PE in POPC) may dwell to a slight extent in a negatively charged patch before diffusing to the next one. However, data as a function of pH (See Fig. S14) suggest this is a rather modest effect. Indeed, the fraction of NBD-PE in the lower leaflet only increases from 0.78 to 0.85 upon raising the pH from 5.0 to 8.5. Therefore, the preference of zwitterionic PE lipids for deprotonated sites should only be marginal.

It should further be noted that hydrated borosilicate/silica surfaces present a mixed population of protonated and deprotonated silanols, and some spatial heterogeneity in local surface charge is expected. Moreover, in the presence of dissolved NaCl, the Na^+ ions work to screen the electrostatic repulsion. As such, local heterogeneity in charge should play only a minor role in lipid distribution.

Lipid Flipping Rate Comparison

The unzipping assay affords a means to determining lipid flip-flop rates lipid in supported bilayers. Using this method, the half-time for NBD-PC translocation in SLBs was measured to be 5.1 ± 0.7 h (Table 1). For comparison, lipid flip-flop in vesicles is generally slower. Half-times for spin-labeled lipids in vesicles have been reported to be on the order of ~7 h at 30 °C.[13] The relatively faster flip-flop rate in SLBs likely arises from the membrane edges or defects that facilitate interleaflet exchange without requiring the polar headgroup to traverse the full hydrophobic core (Fig. 4b). In contrast, lipid translocation in living cell membranes can occur substantially faster (~50 min in red blood cells)[14] due to active, protein-mediated transport processes. It should be noted that most measurements of lipid flip-flop rely on chemically labeled lipids. The presence of fluorescent or spin labels may alter membrane packing or headgroup properties, and therefore potentially affect the intrinsic flipping kinetics.

Additional Figures

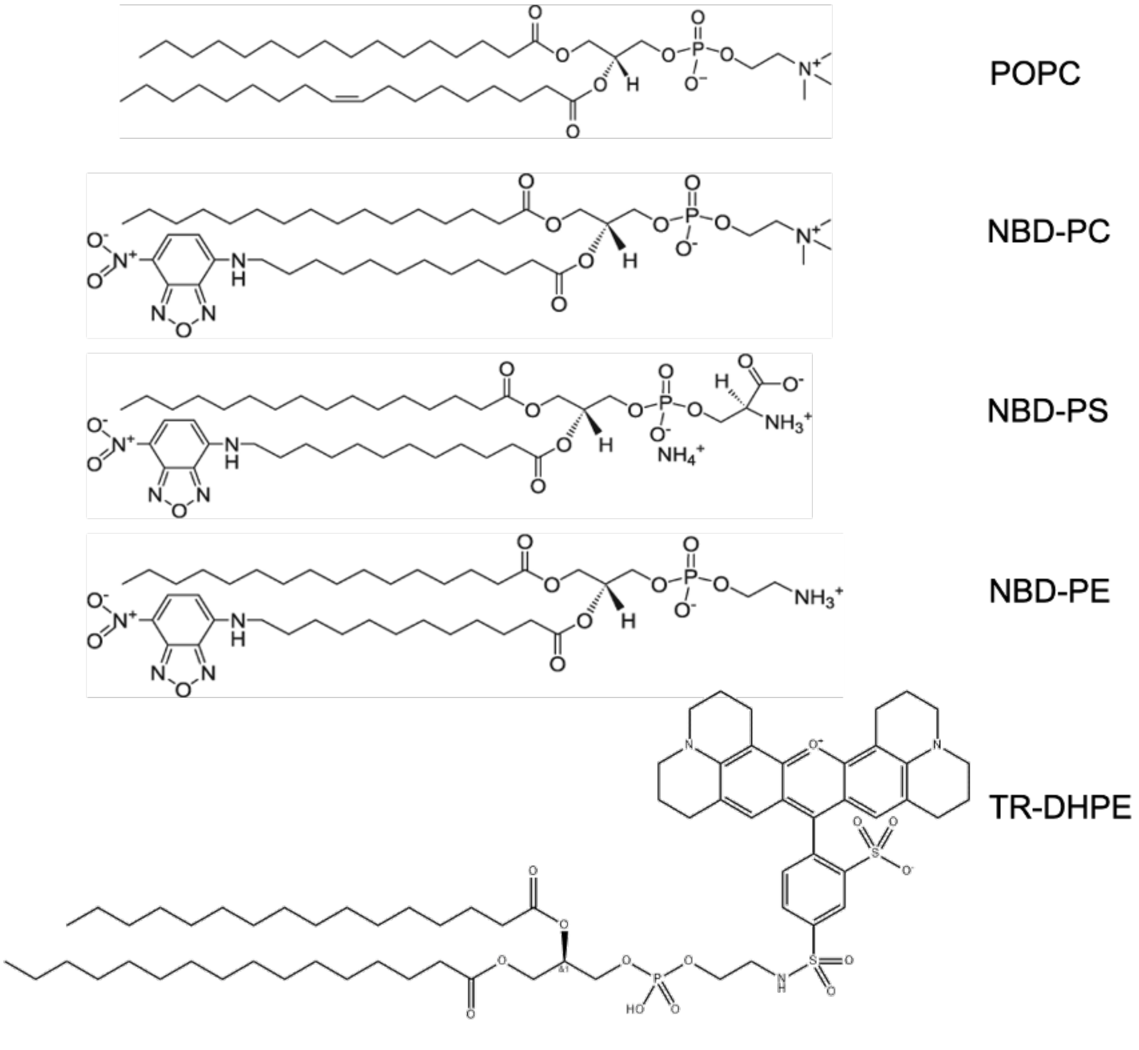

Figure S1. Chemical structure of the lipids used in this study.

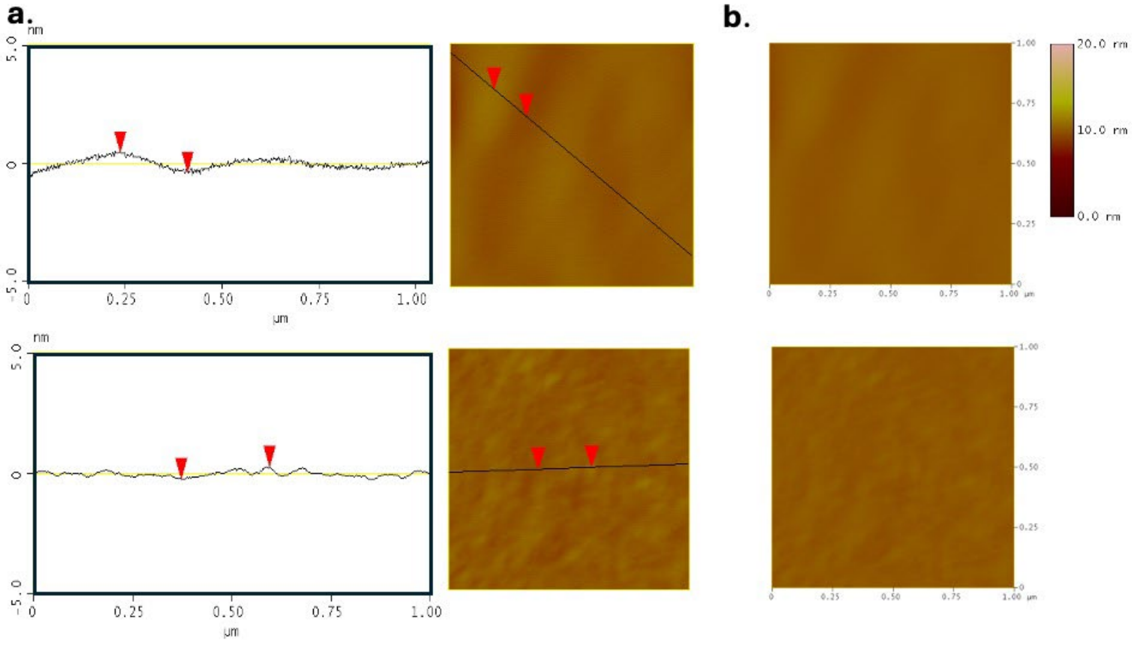

Figure S2: (a) AFM linescan profiles for PDMS (upper panel) and borofloat glass (lower panel). Peaks and troughs are labelled with red arrow. Images to the right of the linescans denote where each linescan was taken (b) 1 µm² images of surface roughness of PDMS (top panel) and glass (bottom panel). The results showed an RMS roughness of 0.17 ± 0.03 nm over a 1 µm² area for PDMS and an RMS roughness of 0.16 ± 0.02 nm over 1 µm² area for glass. Standard deviations were obtained by collecting data over at least three different 1 µm² areas.

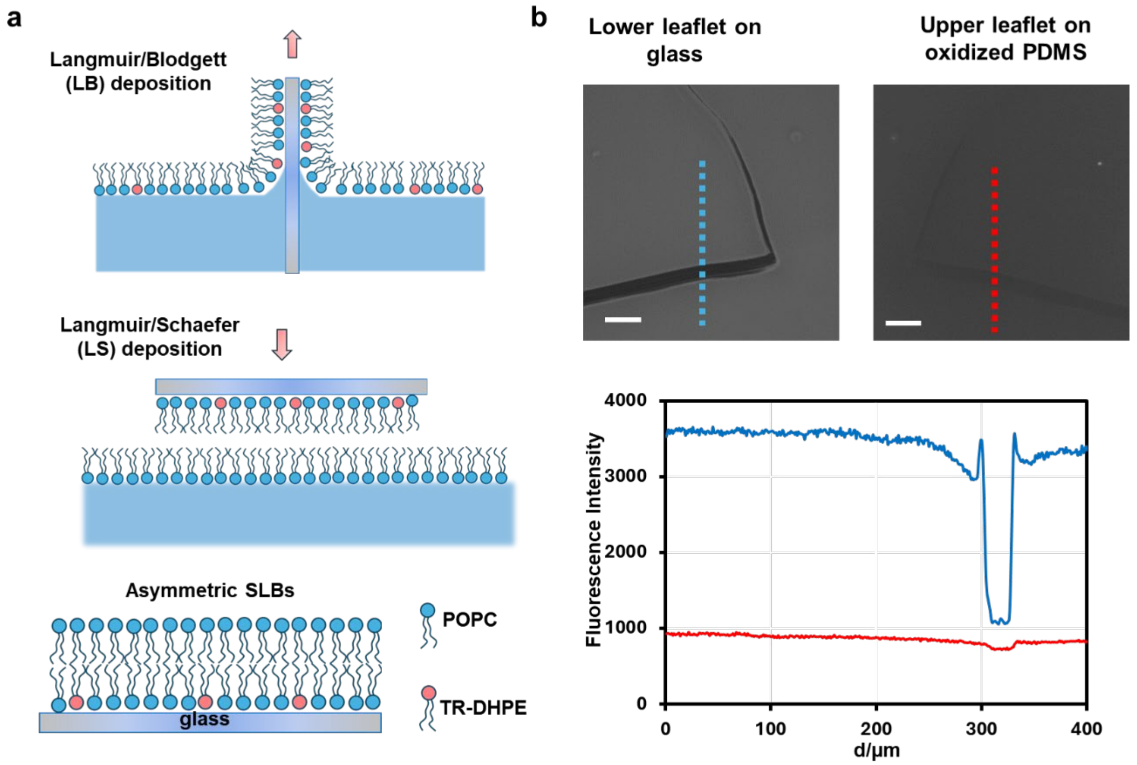

Figure S3. (a) Schematic illustration of an asymmetric supported lipid bilayers formed via the Langmuir-Blodgett/Langmuir-Schaefer method.[5] The lower leaflet contained 0.3 mol% TR-DHPE (red lipid) and 99.7 mol% POPC while the upper leaflet only had POPC. (b)Fluorescence images of a decoupled asymmetric lipid bilayer and the corresponding intensity profile from the two leaflets, indicating that the unzipping assay did not transfer a significant concentration of TR-DHPE from the lower (blue) to the upper (red) leaflet during unzipping. The scale bars are 100µm.

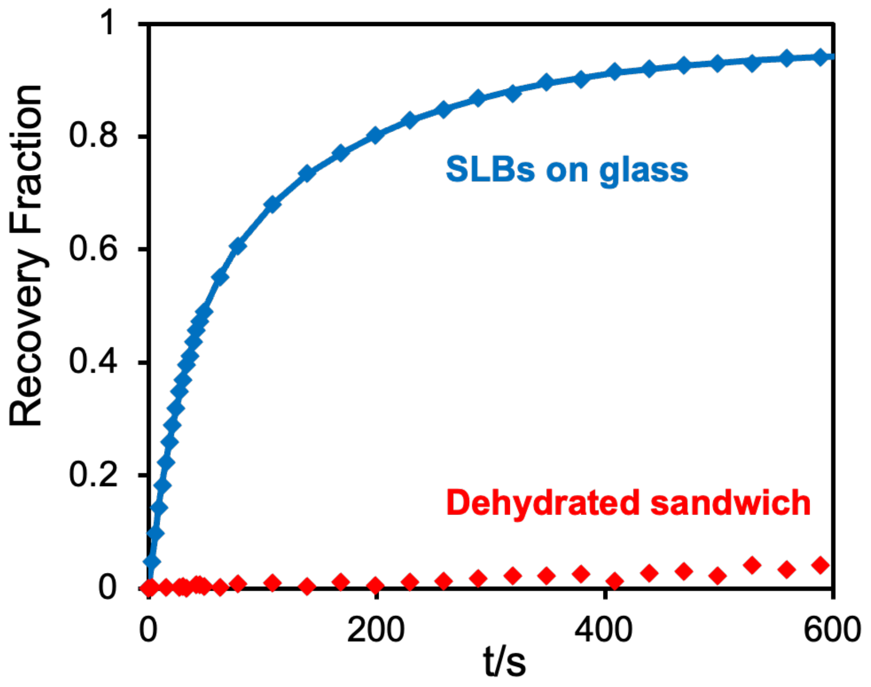

Figure S4. FRAP curve for SLBs (blue) and the dehydrated sandwich (red) after conformal contact with a PDMS slab was made. Lipid composition: 99.7 mol% POPC and 0.3 mol% TR-DHPE.

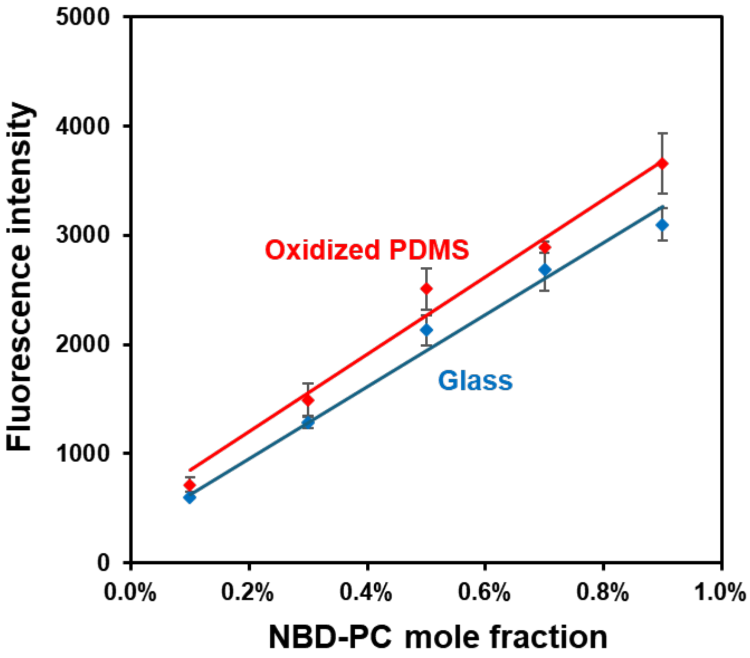

Figure S5. Fluorescence intensity calibration curve for NBD-PC on glass and oxidized PDMS. Fluorescence intensity of POPC lipid monolayer containing 0.1, 0.3, 0.5, 0.7 and 0.9 mol% NBD-PC on glass (blue) and oxidized-PDMS (red) were measured under a fluorescence microscope. Each measurement was repeated 3 times.

a

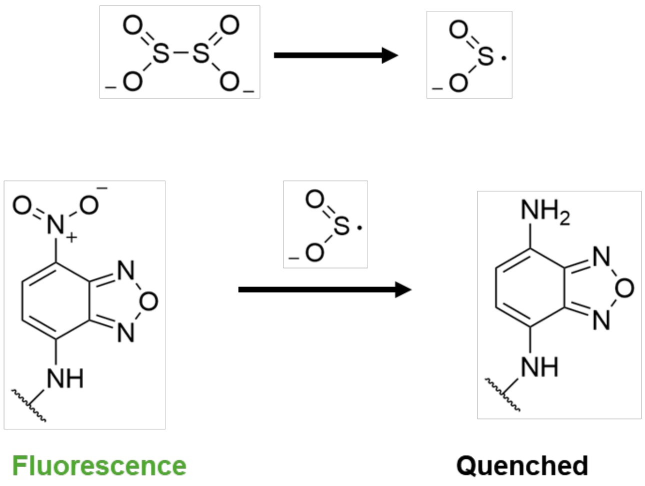

b

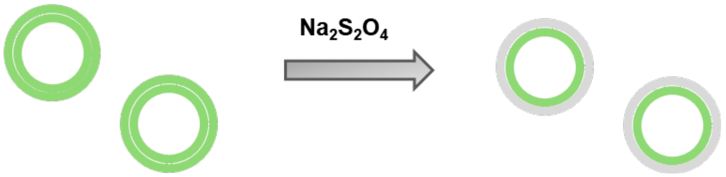

Figure S6. (a) Mechanism for NBD quenching by sodium dithionite. (b)Schematic illustration showing the quenching of the outer leaflet of the vesicles by sodium dithionite.

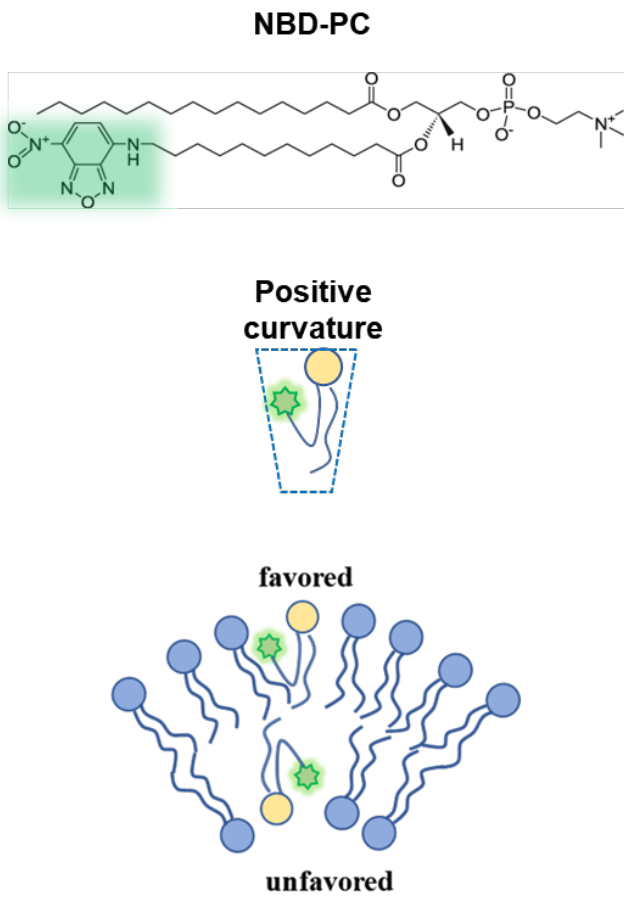

Figure S7. Schematic diagram of the NBD tail-labeled PC lipid. The lower schematic depicts the idea that the covalently-attached dye label can loop around (or snorkel) toward the headgroup. This yields positive intrinsic curvature, which favors the upper leaflet over the lower one.

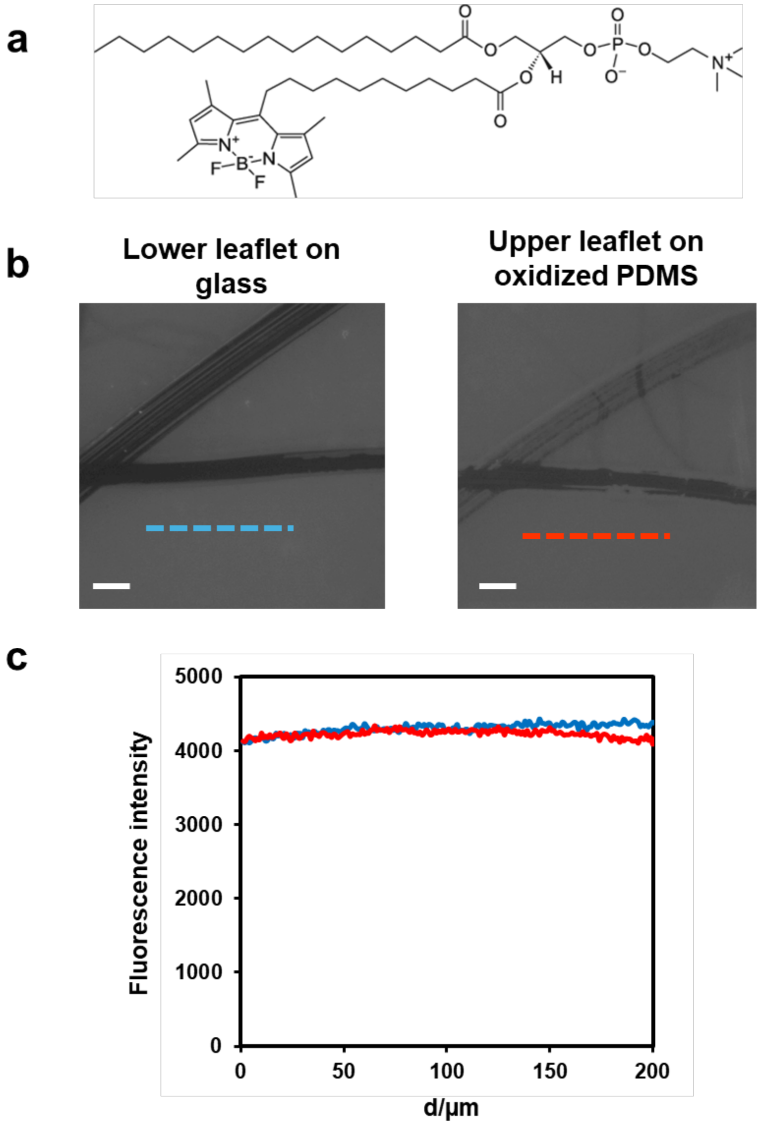

Figure S8. (a) Chemical structure TopFluor-PC. (b) Fluorescence images of a decoupled SLB containing 99.5 mol% POPC and 0.5 mol% TopFluor-PC. (c) Fluorescence intensity profiles come from the dashed lines in (b). Scale bars: 50μm.

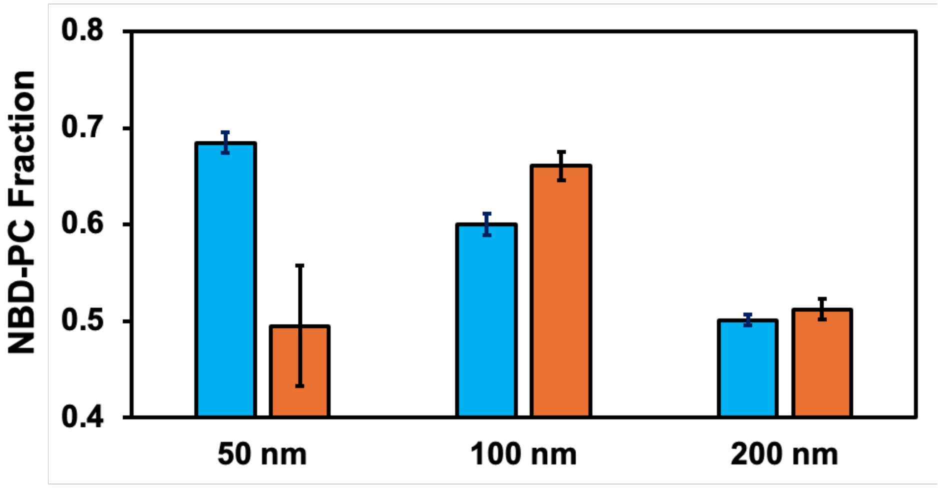

Figure S9: Lower leaflet fractions on glass supports and outer leaflet fractions from vesicles with 50, 100 and 200 nm diameters. Blue bars: outer leaflet fraction in vesicles measure with a dithionite assay; Orange bars: lower leaflet fractions from SLBs measured after unzipping by fluorescence microscopy. The error bars represent standard deviations from four data sets.

As expected, 50 nm vesicles displayed the highest level of lipid vesicle symmetry (68 ± 1% NBD-PC in the outer leaflet, blue bar). The 100 nm vesicles had 60 ± 1% NBD-PC in the outer leaflet (blue bar), while the 200 nm vesicle had no detectable asymmetry (50 ± 0% NBD-PC in the outer leaflet, blue bar). As described in the main text, the NBD-PC in 100 nm vesicle partitioned such that 66 ± 1% NBD-PC was in the inner leaflet after vesicle fusion on the planar glass support. By contrast, the concentration of NBD-PC was only 51 ± 1% in the lower leaflet from 200 nm vesicles. This result is consistent with one's expectation that larger vesicle with less local curvature yield both less vesicle and bilayer asymmetry.

By contrast, 50 nm vesicles gave rise to a more complex outcome. Namely, the highly asymmetric vesicles yielded SLBs with highly irreproducible fractions of NBD-PC in the lower leaflet. In this case the orange bar and error bar comes from four separate trials with 41%, 57%, 43%, and 53% fractions in the lower leaflet. The error bar associated with this is ± 0.07, which is far larger than the lower leaflet fraction error bars from 100 nm and 200 nm vesicles. Such results are consistent with studies on supported bilayer formation by Johnson and Boxer.[15] In their paper, these authors showed that sufficiently small vesicles (50 nm and 30 nm) would not directly fuse to a glass substrate. Instead, they would fuse with each other first at the glass substrate/water interface and only then go on to fuse to the glass substrate. See Fig. S10 for a schematic diagram of this mechanism.

The data shown in Fig. S9 is consistent with the idea that vesicle-vesicle fusion at the interface scrambles lipid asymmetry. Moreover, Johnson and Boxer showed that nascently formed surface-adsorbed vesicles can have a variety of different sizes, which may mean different degrees of mixing, which would be consistent with the larger error bars measured here.

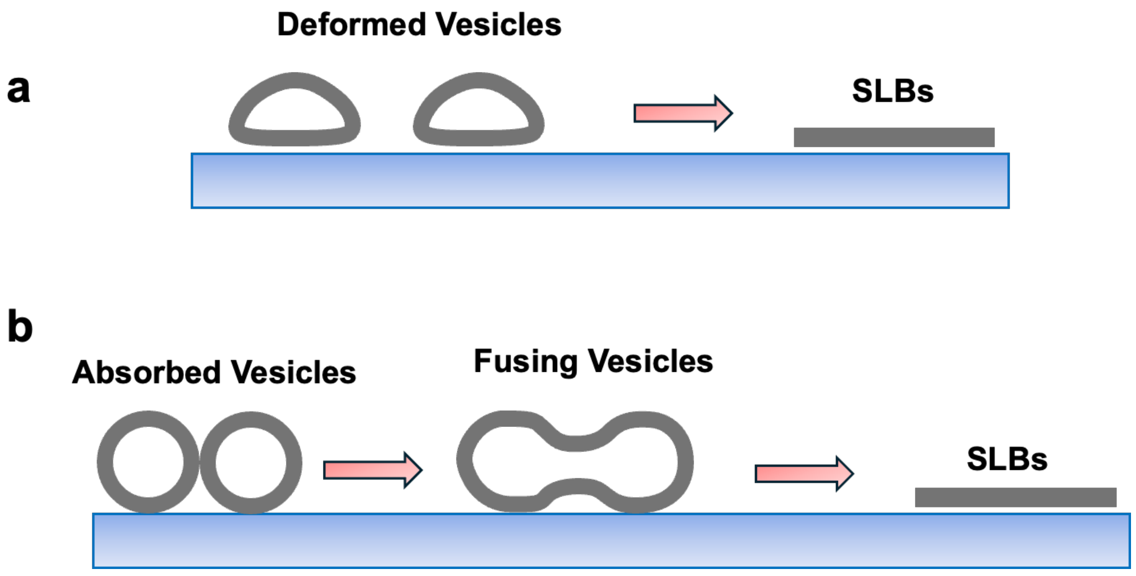

Figure S10. Schematic illustration of two modes of vesicles fusion. (a) Individual small vesicles absorbed onto the substrate followed by vesicle rupture. and fusion. (b) Vesicle-vesicle fusion occurs at the substrate surface, which scrambles and equilibrates the inner and outlet lipid leaflet lipid populations.

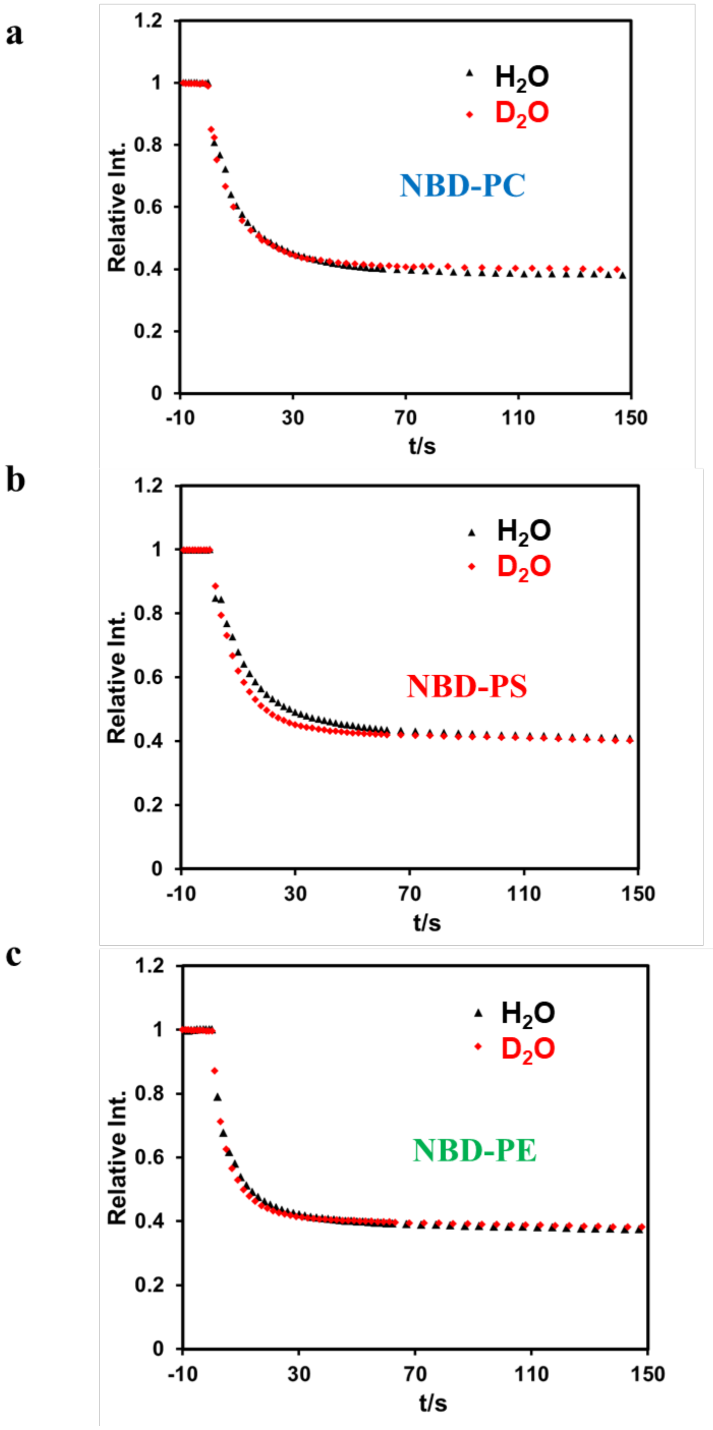

Figure S11. Dithionite quenching curves for lipid vesicles containing 99.5 mol% POPC and 0.5 mol% NBD labeled lipid in H_2O and D_2O buffer. Buffer: 20mM HEPES, 50 mM NaCl, pH 7.4 or pD 7.40. Vesicle size: 100 nm.

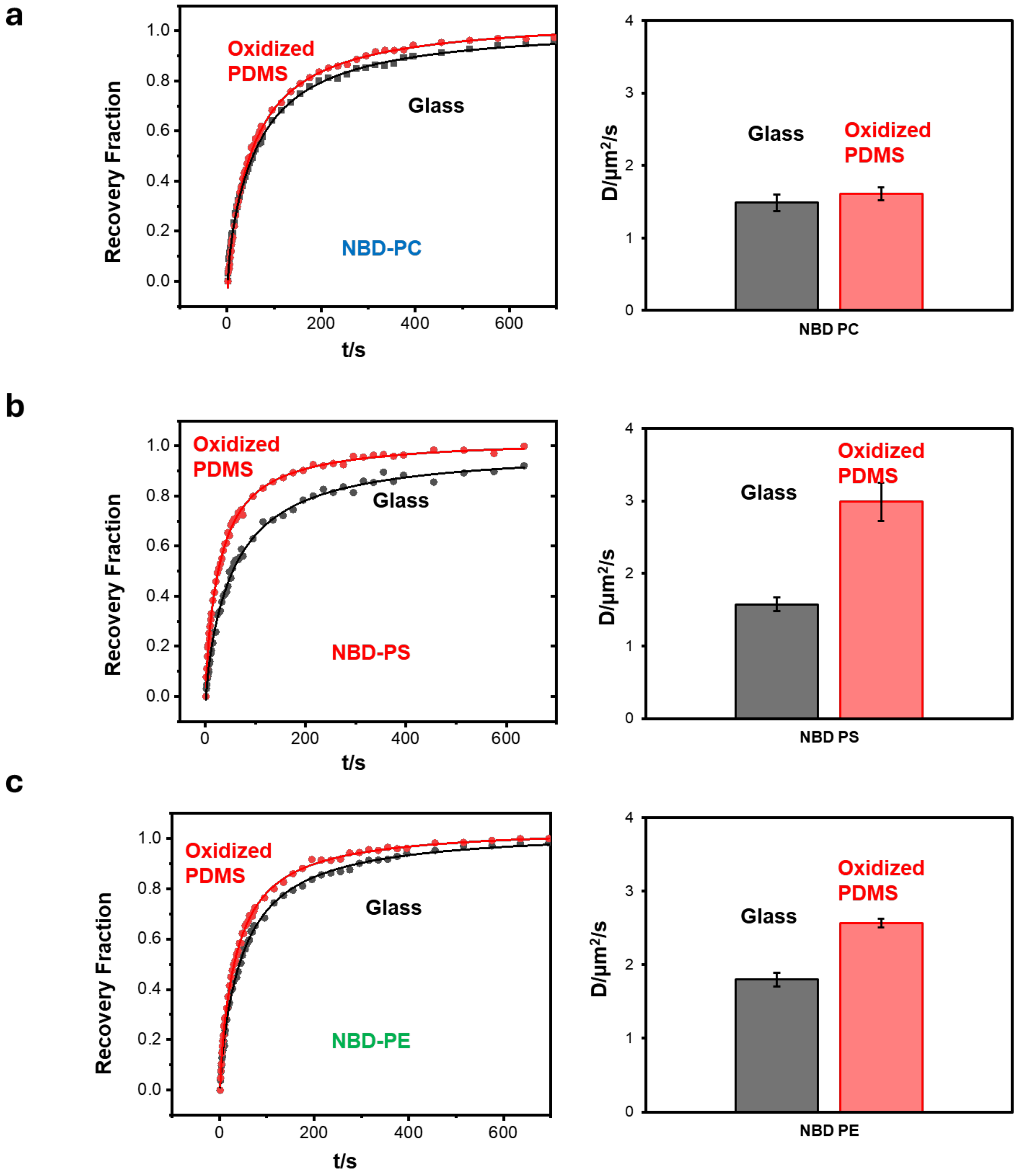

Figure S12. FRAP curves and diffusion coefficients for SLBs containing 99.5 mol% POPC and 0.5 mol% NBD-labeled lipids supported on glass and oxidized PDMS substrates, respectively. Buffer: 20mM HEPES, 50 mM NaCl, pH 7.4. (a) NBD-PC; (b) NBD-PS; (c) NBD-PE.

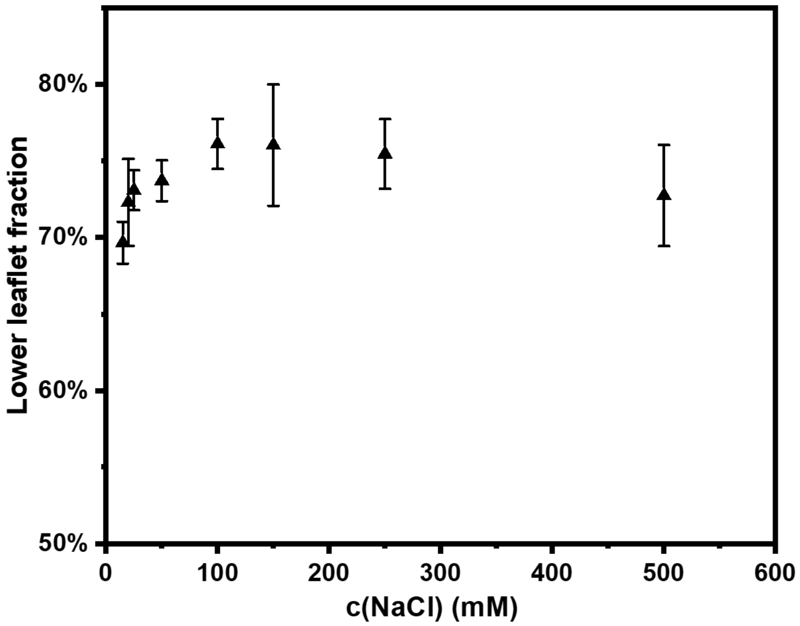

Figure S13. Initial lower leaflet fraction of NBD-PS as a function of NaCl concentration during bilayer formation via vesicle fusion. Buffer: 20 mM HEPES and varied NaCl at pH 7.4.

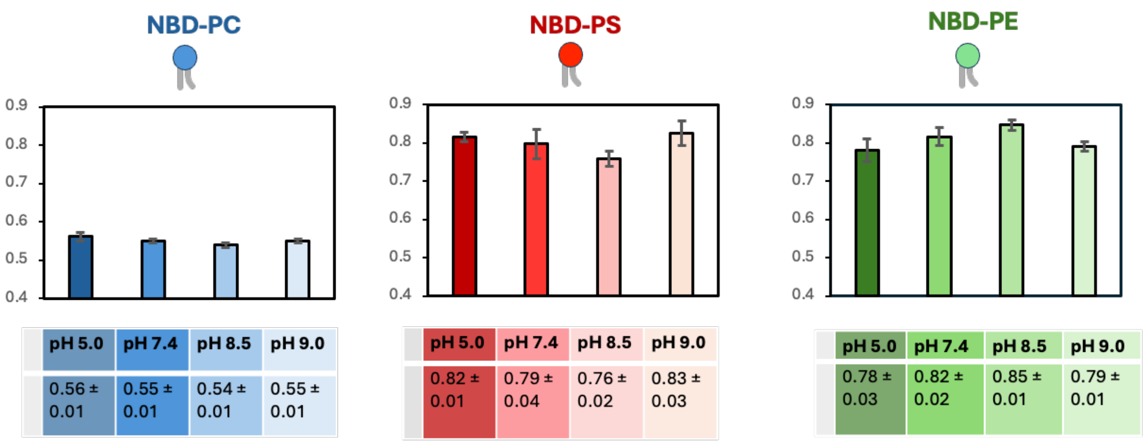

Figure S14. Lower leaflet fraction of NBD-PE (green), NBD-PC (blue), NBD-PS (red) by unzipping bilayer immediately following bilayer formation. These unzippings were done in pH 5.0, 7.4, 8.5, 9.0, (left to right on each graph, respectively). Bilayers were prepared with 99.5% POPC and 0.5% NBD-lipid in 70 mM NaCl using NaOH and HCl to modulate pH. Error bars represent standard deviation from four data sets.

The choline moiety on NBD-PC is not capable of donating a hydrogen bond to the surface. Consistent with this, NBD-PC shows little pH dependence from pH 5.0 to 9.0, supporting the idea that the more buried phosphate group does not play a significant role in lower leaflet enrichment. It should be noted that buffer was not used to perform experiments at varying pH as different buffers can influence the properties of the bilayer. In that respect, it is curious that the lower leaflet fraction of NBD-PC is consistently lower in the absence of 20 mM HEPES (Fig. S14) than in its presence (Fig. 3a). This is consistent with reports that HEPES can interact with membranes and reduce the effective bilayer rigidity.[16] In other words, without HEPES, the bilayer is effectively stiffer during fusion, which would be expected to reduce bilayer deformation and the amount of NBD-PC that can be driven into the lower leaflet during vesicle fusion (Fig. S14).

Next, the pH dependence of NBD-PS was comparatively modest and did not follow a simple trend (lower leaflet fractions of 82 ± 1%, 79 ± 4%, 76 ± 2%, and 83 ± 3% at pH 5.0, 7.4, 8.5, and 9.0, respectively). This muted behavior may reflect competing influences with opposite pH trends. For instance, the PS amine may favor stronger interactions with the more deprotonated surface as pH increases. However, PS also carries an additional carboxylate, increasing the net anionic character of the headgroup and potentially disfavoring close approach to a negatively charged surface at higher pH. In addition, PS headgroups can engage in lipid-lipid headgroups interactions that may compete with lipid-surface interactions. Taken together, these competing effects make the pH response of PS lipids harder to attribute to any single mechanism and likely explain the weaker overall pH sensitivity of PS compared with PE.

Finally, the lower leaflet fraction of NBD-PE increases from pH 5.0 to pH 8.5, then decreases at pH 9.0. This is broadly what one would expect if PE headgroups are stabilized in the lower leaflet by hydrogen bonding to the hydrated glass surface. As pH rises, the surface silanols become progressively deprotonated, which can modestly strengthen interactions with protonated amines (both by increasing hydrogen bond acceptor character and by favorable local electrostatics). At pH

9.0, partial deprotonation of the PE amine would reduce its ability to engage in these interactions, consistent with the observed drop in the lower leaflet fraction.

Overall, the pH results are consistent with the salt screening experiments in Fig. 5 in the main paper. Namely, the influence of electrostatics is of less importance than H-bonding, when sufficient salt is present.

References:

(1) Cremer, P. S.; Boxer, S. G. Formation and Spreading of Lipid Bilayers on Planar Glass Supports. *J. Phys. Chem. B* **1999**, *103* (13), 2554–2559.

(2) Sun, S.; Liu, C.; Rodriguez Melendez, D.; Yang, T.; Cremer, P. S. Immobilization of Phosphatidylinositides Revealed by Bilayer Leaflet Decoupling. *J. Am. Chem. Soc.* **2020**, *142* (30), 13003–13010.

(3) Soumpasis, D. M. Theoretical Analysis of Fluorescence Photobleaching Recovery Experiments. *Biophys. J.* **1983**, *41* (1), 95–97.

(4) Goodchild, J. A.; Walsh, D. L.; Laurent, H.; Connell, S. D. PDMS as a Substrate for Lipid Bilayers. *Langmuir* **2023**, *39* (31), 10843–10854.

(5) Diaz, A. J.; Albertorio, F.; Daniel, S.; Cremer, P. S. Double Cushions Preserve Transmembrane Protein Mobility in Supported Bilayer Systems. *Langmuir* **2008**, *24* (13), 6820–6826.

(6) Castellana, E. T.; Cremer, P. S. Solid Supported Lipid Bilayers: From Biophysical Studies to Sensor Design. *Surf. Sci. Rep.* **2006**, *61* (10), 429–444.

(7) Schrader, A. M.; Monroe, J. I.; Sheil, R.; Dobbs, H. A.; Keller, T. J.; Li, Y.; Jain, S.; Shell, M. S.; Israelachvili, J. N.; Han, S. Surface Chemical Heterogeneity Modulates Silica Surface Hydration. *Proc. Natl. Acad. Sci.* **2018**, *115* (12), 2890–2895.

(8) Behrens, S. H.; Grier, D. G. The Charge of Glass and Silica Surfaces. *J. Chem. Phys.* **2001**, *115* (14), 6716–6721.

(9) Hahn, S. H.; Van Duin, A. C. T. Surface Reactivity and Leaching of a Sodium Silicate Glass under an Aqueous Environment: A ReaxFF Molecular Dynamics Study. *J. Phys. Chem. C* **2019**, *123* (25), 15606–15617.

(10) Brown, M. A.; Goel, A.; Abbas, Z. Effect of Electrolyte Concentration on the Stern Layer Thickness at a Charged Interface. *Angew. Chem. Int. Ed.* **2016**, *55* (11), 3790–3794.

(11) White, R. J.; Ervin, E. N.; Yang, T.; Chen, X.; Daniel, S.; Cremer, P. S.; White, H. S. Single Ion-Channel Recordings Using Glass Nanopore Membranes. *J. Am. Chem. Soc.* **2007**, *129* (38), 11766–11775.

(12) Schaut, R. A.; Lobello, R. A.; Mueller, K. T.; Pantano, C. G. Characterization of Boroaluminosilicate Glass Surface Structures by B *K*-Edge NEXAFS. *J. Non-Cryst. Solids* **2011**, *357* (19), 3416–3423.

(13) McConnell, H. M.; Kornberg, R. D. Inside-Outside Transitions of Phospholipids in Vesicle Membranes. *Biochemistry* **1971**, *10* (7), 1111–1120.

(14) Morrot, G.; Herve, P.; Zachowski, A.; Fellmann, P.; Devaux, P. F. Aminophospholipid Translocase of Human Erythrocytes: Phospholipid Substrate Specificity and Effect of Cholesterol. *Biochemistry* **1989**, *28* (8), 3456–3462.

(15) Johnson, J. M.; Ha, T.; Chu, S.; Boxer, S. G. Early Steps of Supported Bilayer Formation Probed by Single Vesicle Fluorescence Assays. *Biophys. J.* **2002**, *83* (6), 3371–3379.

(16) Johnson, M. A.; Seifert, S.; Petrache, H. I.; Kimble-Hill, A. C. Phase Coexistence in Single-Lipid Membranes Induced by Buffering Agents. *Langmuir* **2014**, *30* (33), 9880–9885.
